# Sesquiterpenoids From the Antarctic Fungus *Pseudogymnoascus* sp. HSX2#-11

**DOI:** 10.3389/fmicb.2021.688202

**Published:** 2021-06-11

**Authors:** Ting Shi, Xiang-Qian Li, Li Zheng, Ya-Hui Zhang, Jia-Jia Dai, Er-Lei Shang, Yan-Yan Yu, Yi-Ting Zhang, Wen-Peng Hu, Da-Yong Shi

**Affiliations:** ^1^State Key Laboratory of Microbial Technology, Institute of Microbial Technology, Shandong University, Qingdao, China; ^2^Laboratory for Marine Drugs and Bioproducts of Qingdao National Laboratory for Marine Science and Technology, Qingdao, China; ^3^Key Laboratory of Marine Eco-Environmental Science and Technology, First Institute of Oceanography, Ministry of Natural Resources, Qingdao, China; ^4^Laboratory for Marine Ecology and Environmental Science, Qingdao Pilot National Laboratory for Marine Science and Technology, Qingdao, China; ^5^Key Laboratory of Marine Drugs, The Ministry of Education of China, School of Medicine and Pharmacy, Ocean University of China, Qingdao, China; ^6^Laboratory for Marine Drugs and Bioproducts, Qingdao National Laboratory for Marine Science and Technology, Qingdao, China; ^7^State Key Laboratory of Pharmaceutical Biotechnology, School of Life Sciences, Nanjing University, Nanjing, China

**Keywords:** Antarctic fungus, *Pseudogymnoascus* sp. HSX2#-11, sesquiterpenoids, steroids, cytotoxicity, antibacterial activity

## Abstract

The fungal strains *Pseudogymnoascus* are a kind of psychrophilic pathogenic fungi that are ubiquitously distributed in Antarctica, while the studies of their secondary metabolites are infrequent. Systematic research of the metabolites of the fungus *Pseudogymnoascus* sp. HSX2#-11 led to the isolation of six new tremulane sesquiterpenoids pseudotremulanes A–F (**1**–**6**), combined with one known analog 11,12-epoxy-12β-hydroxy-1-tremulen-5-one (**7**), and five known steroids (**8**–**12**). The absolute configurations of the new compounds (**1**–**6**) were elucidated by their ECD spectra and ECD calculations. Compounds **1**–**7** were proved to be isomeride structures with the same chemical formula. Compounds **1**/**2**, **3**/**4**, **1**/**4**, and **2**/**3** were identified as four pairs of epimerides at the locations of C-3, C-3, C-9, and C-9, respectively. Compounds **8** and **9** exhibited cytotoxic activities against human breast cancer (MDA-MB-231), colorectal cancer (HCT116), and hepatoma (HepG2) cell lines. Compounds **9** and **10** also showed antibacterial activities against marine fouling bacteria *Aeromonas salmonicida*. This is the first time to find terpenoids and steroids in the fungal genus *Pseudogymnoascus*.

## Introduction

Tremulanes, a family of sesquiterpenoids with characteristic structures of 5/7 fused bicyclic system, were rarely discovered in nature until 2015 (Guo et al., 2016). However, from 2016 to 2020, about 60 tremulane derivatives were found (Guo et al., 2016; Isaka et al., 2016; Wu, 2016; Chen et al., 2017, 2018; Cong et al., 2020; Wang et al., 2017, 2020; Ding et al., 2018, 2019, 2020a,b; Zhou et al., 2018; Duan et al., 2019; Wu et al., 2019, 2020; He et al., 2020; Lee et al., 2020; Shi et al., 2020; Sun C.-T. et al., 2020), and the number is twice as many as before. Most of them were isolated from the cultures of the basidiomycetes *Irpex lacteus* (Chen et al., 2018, 2020; Ding et al., 2018, 2019, 2020a,b; Zhou et al., 2018; Duan et al., 2019; Wu et al., 2019; Shi et al., 2020; Sun C.-T. et al., 2020; Wang et al., 2020). All the tremulanes isolated from 2016 to 2020 were derived from fungi, except one derivative, which was obtained from a traditional Chinese medicine tabasheer (Wu, 2016). Some of them were discovered to have different bioactivities, such as tremutin A with the inhibition of the lipopolysaccharide-induced proliferation of B lymphocyte cells (Wang et al., 2020), and 5-demethyl conocenol C showed antifungal activities (Wu et al., 2019).

**FIGURE 1:**
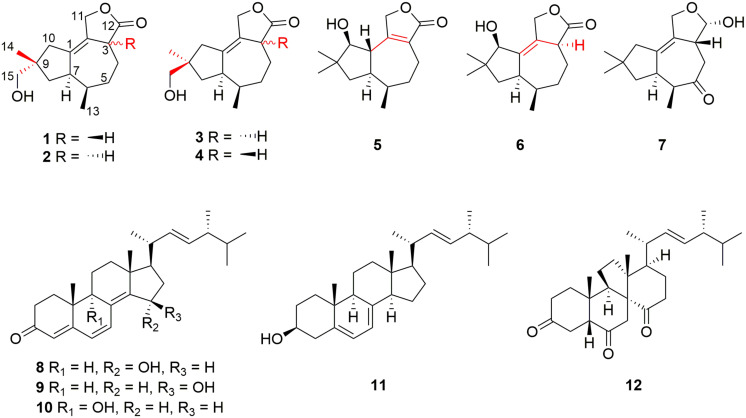
Structures of compounds **1**–**12**.

The extreme environments of Antarctica, including cold, dry climate and intense solar radiations, have nurtured a number of unique microbial resources (Cong et al., 2020). It has been proved that Antarctic microorganisms, especially fungi, have the potential capacity to produce novel secondary metabolites to adapt to the harsh environments (Kwon et al., 2017; Rusman et al., 2018; Yu et al., 2019; Sun C. et al., 2020). *Pseudogymnoascus* are known as a kind of psychrophilic pathogenic fungi with ubiquitous distribution in Antarctica (Rosa et al., 2020; Santos et al., 2020; Martorell et al., 2021). These fungal strains have been proved to have the abilities to produce cold-adapted enzymes to adapt severe cold Antarctic environment (Loperena et al., 2012; Poveda et al., 2018). *Pseudogymnoascus* can be antagonistic fungi against potato scab pathogens from potato field soils (Tagawa et al., 2010) and have been certified to be one of the predominant microbial colonizers in the root endosphere and rhizosphere of turfgrass systems (Xia et al., 2021). The extracts of some of *Pseudogymnoascus* strains exhibit potent bioactivities, such as antimicrobial, herbicidal, and antitumoral activities (Henríquez et al., 2014; Gonçalves et al., 2015; Gomes et al., 2018; Ferrarezi et al., 2019). However, only four studies have been done on the secondary metabolites of the genus *Pseudogymnoascus* until now, as far as we know, and most of the obtained structures focus on polyketides, showing antimicrobial activities (Figueroa et al., 2015; Guo et al., 2019; Fujita et al., 2021; Shi et al., 2021). Rare studies about the secondary metabolites of these fungi enlighten that there is latent space for searching novel compounds. *Pseudogymnoascus* sp. HSX2#-11 was an Antarctic fungus isolated from a soil sample of the Fields Peninsula, which can produce abundant and various secondary metabolites, according to our previous research on the fingerprint spectrum and molecular network of its ethyl acetate extract of the fermentation broth (Shi et al., 2021). Further chemical investigation resulted in the isolation and identification of six new tremulane sesquiterpenoids, pseudotremulanes A–F (**1**–**6**), together with one known analog 11,12-epoxy-12β-hydroxy-1-tremulen-5-one (**7**; Zhou et al., 2008), and five known steroids, ganodermasides A (**8**), B (**9**), and D (**10**; Weng et al., 2010, 2011), ergosterol (**11**; Feng et al., 2010), and dankasterone B (**12**; Amagata et al., 2007; Figure 1). Compounds **8** and **9** exhibited cytotoxicities against human breast cancer cell line MDA-MB-231, colorectal cancer cell line HCT116, and hepatoma cell line HepG2 (Table 3). Compounds **9** and **10** showed antibacterial activity against marine fouling bacteria *Aeromonas salmonicida*. Here, we address the isolation, structure elucidation, and biological activity evaluation of the isolated compounds.

## Experimental Section

### General Experimental Procedures

Optical rotations were measured on a JASCO P-1020 digital polarimeter (JASCO, Japan). The UV spectrum was recorded using an Implen Gmbh NanoPhotometer N50 Touch (Implen, Germany). ECD spectra were obtained on a Jasco J-815-150S circular dichroism spectrometer (JASCO, Japan). NMR spectra were recorded on a Bruker AVANCE NEO (Bruker, Switzerland) at 600 MHz for ^1^H and 150 MHz for ^13^C in CDCl_3_. Chemical shifts δ were recorded in ppm using TMS as the internal standard. HR-APCI-MS spectra were measured on a Thermo Scientific LTQ Orbitrap XL spectrometer (Thermo Fisher Scientific, Bremen, Germany). HPLC separation was performed using a Hitachi Primaide Organizer Semi-HPLC system (Hitachi High Technologies, Tokyo, Japan) coupled with a Hitachi Primaide 1430 photodiodearray detector (Hitachi High Technologies, Tokyo, Japan). A Kromasil C_18_ semi-preparative HPLC column (250 × 10 mm, 5 μm; Eka Nobel, Bohus, Sweden) was used. Silica gel (200–300 mesh; Qingdao Marine Chemical Group Co., Qingdao, China) and Sephadex LH-20 (Amersham Biosciences Inc., Piscataway, NJ, United States) were used for column chromatography (CC). Precoated silica gel GF254 plates (20 × 20 cm, Yantai Zifu Chemical Group Co., Yantai, China).

### Fungal Materials

The soil samples were collected in ice-free areas (about 10 cm underground) of the Fields Peninsula using sterile spatulas and sterilized WhirlPak bags (Sigma-Aldrich, United States), and were transported to the lab in sealed foam package with dry ice added by airplane, at the Chinese 35th Antarctic expedition in 2019. The fungus *Pseudogymnoascus* sp. HSX2#-11 was isolated from a soil sample from Fields Peninsula. The strain was deposited at −80°C in the State Key Laboratory of Microbial Technology, Institute of Microbial Technology, Shandong University, Qingdao, China.

**FIGURE 2:**
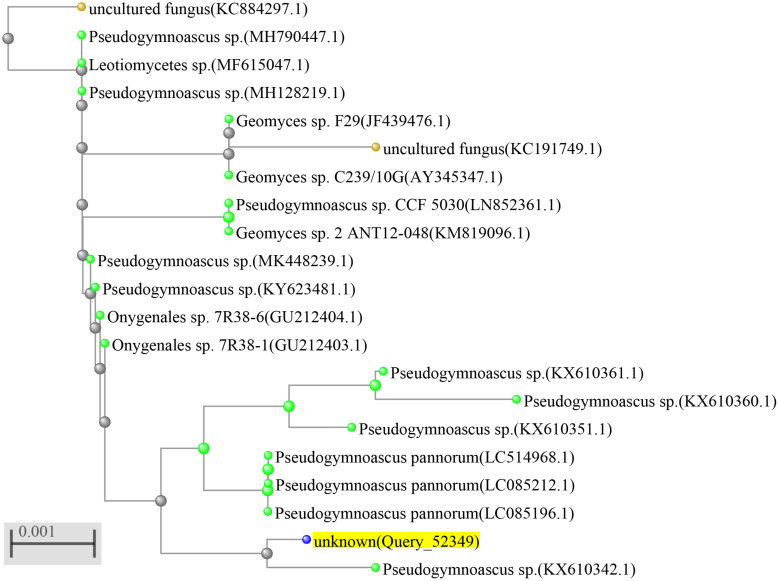
Phylogenetic tree of the fungus *Pseudogymnoascus* sp. HSX2#-11.

The identification of the fungal strain HSX2#-11 was conducted by the analysis of the 28S rRNA gene sequence. The fresh fungal mycelium (about 1.00 mg) was dispersed in a 50 μl lysis buffer for the microorganisms to direct PCR (Takara, Cat# 9164), saved in metal bath (Yooning, China) at 100°C for 30 min to extract its genomic DNA as the template DNA. The PCRs were performed in a final volume of 50 μl, which was composed of the template DNA (3 μl), ITS1 (1 μl), ITS4 (1 μl), PrimeSTAR^®^ Max DNA Polymerase (25 μl, Takara, Cat# R045A), and ultrapure water (20 μl), under the following procedures: (1) initial denaturation at 98°C for 5 min; (2) denaturation at 98°C for 30 s; (3) annealing at 55°C for 30 s; (4) extension at 72°C for 1 min; and (5) final extension at 72°C for 10 min. Steps 2–4 were repeated 30 times. The PCR products were then submitted for sequencing (BGI, China) with the primers ITS1 and ITS4. The sequence of HSX2#-11 was searched in the NCBI nucleotide collection database through the BLAST program. The phylogenetic tree of the top 20 most similar to this fungal sequence identified the strain HSX2#-11 as a *Pseudogymnoascus* sp. (Figure 2), with GenBank (NCBI) accession number MT367223.1.

### Extraction and Isolation

The fungal strain *Pseudogymnoascus* sp. HSX2#-11 was fermented in a PDA liquid medium in 200 Erlenmeyer flasks (300 ml in each 1,000-ml flask) at 16°C in air condition room for 45 days. The culture (60 L) was filtered to separate the broth from the mycelia. Then the mycelia were extracted three times with EtOAc (3 × 4,000 ml) and then repeatedly extracted with CH_2_Cl_2_–MeOH (v/v, 1:1) three times (3 × 4,000 ml). The broth was extracted repeatedly with EtOAc (3 × 60 L) to get the EtOAc layer. All the extracts were combined and then evaporated to dryness under reduced pressure to afford a residue (71.5 g). The residue was subjected to vacuum liquid chromatography on silica gel using step gradient elution with EtOAc–petroleum ether (PE; 0–100%) and then with MeOH–EtOAc (0–100%) to afford eight fractions (Fr.1–Fr.8). Fr.2 was the pure compound **11** (89.7 mg). Fr.3 was first subjected to the gradient elution of ODS CC with MeOH in H_2_O (10–100%) and then purified by using semipreparative HPLC on an ODS column (Kromasil C_18_, 250 × 10 mm, 5 μm, 2 ml/min) eluted with 85% MeOH–H_2_O to give compound **12** (2.7 mg). Fr.4 was isolated by CC on Sephadex LH-20 eluted with CH_2_Cl_2_–MeOH (v/v, 1:1) to afford two fractions (Fr.4.1, Fr.4.2). Fr.4.1 was subjected to silica gel CC eluting with EtOAc–PE (0–50%) to get three fractions (Fr.4.1.1–4.1.3). Fr.4.1.1 was first purified by HPLC eluted with 60% MeOH–H_2_O to give compound **7** (1.9 mg), and then purified by HPLC eluted with 40% MeCN–H_2_O to afford **5** (0.1 mg) and **6** (0.2 mg). Fr.4.1.2 was subjected on HPLC eluting with 35% MeCN–H_2_O to give **1** (0.7 mg) and **2** (0.7 mg). Fr.4.1.3 was separated on HPLC eluting with 30% MeCN–H_2_O to get **3** (0.6 mg) and **4** (0.5 mg). Fr.4.2 was first separated on silica gel CC eluting with EtOAc–PE (0–50%), and then purified by HPLC eluting with 75% MeOH–H_2_O to gain **8** (13.6 mg), **10** (3.1 mg), and **9** (11.1 mg).

Pseudotremulane A (**1**): colorless oil; [*α*]_D_^20^ + 8.6 (*c* 0.058, MeOH); UV (CH_2_Cl_2_) λ_*max*_ (log ε): 224 (4.90) nm; CD (3.4 mM, MeOH) λ_*max*_ (Δε) 204 (+9.51), 230 (−4.31) nm; ^1^H and ^13^C NMR data, see Tables 1, 2; HR-APCI-MS m/z 251.1641 [M + H]^+^ (calcd for C_15_H_23_O_3_, 251.1642).

**TABLE 1:** ^1^H NMR data of compounds **1**–**6** in CDCl_3_ at 600 MHz.

**No.**	**1**	**2**	**3**	**4**	**5**	**6**
1					2.71, t (10.5)	
3	3.03–2.99, m	3.14–3.08, m	3.15, d (12.1)	3.03, d (12.2)		3.16, dt (12.1, 2.8)
4	2.23, dd (13.5, 6.1)	1.95, ddt (14.0, 5.3, 2.7)	1.96, ddt (13.3, 5.5, 2.8)	2.25, dd (14.1, 6.2)	2.47, d (16.9)	2.01–1.96, m
	1.75, dd (13.5, 6.2)	1.62, ddd (14.0, 12.5, 2.1)	1.63, ddd (13.3, 12.1, 2.1)	1.88–1.78, m	2.38–2.29, m	1.65, d (13.2)
5	2.05, dd (14.2, 8.5)	2.03–1.98, m	2.04–1.99, m	2.11–2.04, m	1.71–1.62, m	2.05–2.02, m
	1.47, td (14.2, 6.2)	1.77, dt (13.5, 2.7)	1.81–1.79, m	1.49, dt (13.3, 6.2)		1.79, t (13.2)
6	2.11–2.07, m	1.91–1.86, m	1.91, dq (7.0, 3.0)	2.17–2.11, m	2.08–2.00, m	1.96–1.91, m
7	3.10–3.03, m	2.89–2.83, m	2.98–2.92, m	3.16, br s	2.08–2.00, m	2.91–2.86, m
8	1.82–1.77, m	1.82–1.79, m	1.58, t (12.0)	1.60, t (12.0)	1.60–1.56, m	1.69, d (12.0)
	1.41, t (12.5)	1.40, dd (13.3, 10.7)	1.50, dd (12.0, 8.4)	1.42, dd (12.0, 7.3)	1.53–1.50, m	1.46, dd (12.0, 8.1)
10	2.32, d (17.5)	2.13, d (16.1)	2.07, d (15.7)	2.17–2.11, m	3.64, dd (10.5, 6.2)	3.73, s
	1.90, d (17.5)	1.86–1.82, m	1.85–1.81, m	2.11–2.04, m		
11	4.67, d (10.3)	4.75, d (13.2)	4.75, d (13.3)	4.67, d (12.0)	4.91, d (17.8)	5.00, d (13.6)
	4.65, d (10.3)	4.69, d (13.2)	4.68, d (13.3)	4.62, d (12.0)	4.81, d (17.8)	4.88, d (13.6)
13	0.93, d (7.2)	0.88, d (6.9)	0.86, d (7.0)	0.93, d (7.1)	0.96, d (5.8)	0.91, d (7.0)
14	1.11, s	1.12, s	0.92, s	1.06, s	0.95, s	1.07, s
15	3.46, d (10.6)	3.30, d (10.6)	3.52, s	3.49, s	1.07, s	0.82, s
	3.39, d (10.6)	3.26, d (10.6)				

**TABLE 2:** ^13^C NMR data of compounds **1**–**6** in CDCl_3_ at 150 MHz.

**No.**	**1**	**2**	**3**	**4**	**5**	**6**
1	138.5, C	138.8, C	138.4, C	138.0, C	45.0, CH	140.8, C
2	125.0, C	125.6, C	125.8, C	125.2, C	162.9, C	132.6, C
3	43.56, CH	44.5, CH	44.6, CH	43.6, CH	128.8, C	44.7, CH
4	26.5, CH_2_	22.7, CH_2_	22.6, CH_2_	26.5, CH_2_	20.1, CH_2_	22.2, CH_2_
5	33.1, CH_2_	36.9, CH_2_	36.9, CH_2_	33.2, CH_2_	33.5, CH_2_	36.9, CH_2_
6	32.7, CH	31.5, CH	31.5, CH	32.6, CH	32.7, CH	30.7, CH
7	43.59, CH	48.4, CH	47.7, CH	43.2, CH	41.5, CH	47.5, CH
8	40.8, CH_2_	40.1, CH_2_	39.7, CH_2_	40.6, CH_2_	42.3, CH_2_	41.1, CH_2_
9	42.6, C	43.8, C	43.9, C	42.8, C	39.0, C	42.6, C
10	41.2, CH_2_	41.5, CH_2_	41.6, CH2	41.1, CH_2_	83.6, CH	80.1, CH
11	69.20, CH_2_	69.6, CH_2_	69.6, CH2	69.2, CH_2_	71.1, CH_2_	68.9, CH2
12	177.8, C	179.3, C	179.3, C	177.8, C	175.3, C	178.6, C
13	17.5, CH_3_	12.0, CH_3_	12.1, CH_3_	17.7, CH_3_	12.1, CH_3_	12.9, CH_3_
14	24.3, CH_3_	23.6, CH_3_	22.7, CH_3_	23.2, CH_3_	23.9, CH_3_	22.1, CH_3_
15	69.18, CH_2_	68.9, CH_2_	71.2, CH_2_	71.5, CH_2_	29.1, CH_3_	25.7, CH_3_

Pseudotremulane B (**2**): colorless oil; [*α*]_D_^20^ + 13.9 (*c* 0.058, MeOH); UV (CH_2_Cl_2_) λ_*max*_ (log ε): 223 (4.82); CD (3.4 mM, MeOH) λ_*max*_ (Δε) 223 (+4.80) nm; ^1^H and ^13^C NMR data, see Tables 1, 2; HR-APCI-MS m/z 251.1641 [M + H]^+^ (calcd for C_15_H_23_O_3_, 251.1642).

Pseudotremulane C (**3**): colorless oil; [*α*]_D_^20^ + 20.6 (*c* 0.050, MeOH); UV (CH_2_Cl_2_) λ_*max*_ (log ε): 228 (4.48); CD (4.0 mM, MeOH) λ_*max*_ (Δε) 217 (+3.05) nm; ^1^H and ^13^C NMR data, see Tables 1, 2; HR-APCI-MS m/z 251.1642 [M + H]^+^ (calcd for C_15_H_23_O_3_, 251.1642).

Pseudotremulane D (**4**): colorless oil; [*α*]_D_^20^ + 8.5 (*c* 0.042, MeOH); UV (CH_2_Cl_2_) λ_*max*_ (log ε): 223 (4.88); CD (2.4 mM, MeOH) λ_*max*_ (Δε) 205 (+13.51), 232 (−5.56) nm; ^1^H and ^13^C NMR data, see Tables 1, 2; HR-APCI-MS m/z 251.1641 [M + H]^+^ (calcd for C_15_H_23_O_3_, 251.1642).

Pseudotremulane E (**5**): colorless oil; [*α*]_D_^20^−75.0 (*c* 0.008, MeOH); UV (CH_2_Cl_2_) λ_*max*_ (log ε): 224 (5.51); CD (1.2 mM, MeOH) λ_*max*_ (Δε) 222 (−0.29), 247 (+1.29) nm; ^1^H and ^13^C NMR data, see Tables 1, 2; HR-APCI-MS m/z 251.1638 [M + H]^+^ (calcd for C_15_H_23_O_3_, 251.1642).

Pseudotremulane F (**6**): colorless oil; [*α*]_D_^20^ + 7.9 (*c* 0.017, MeOH); UV (CH_2_Cl_2_) λ_*max*_ (log ε): 221 (5.17); CD (6.0 mM, MeOH) λ_*max*_ (Δε) 223 (+4.29) nm; ^1^H and ^13^C NMR data, see Tables 1, 2; HR-APCI-MS m/z 251.1641 [M + H]^+^ (calcd for C_15_H_23_O_3_, 251.1642).

### Cytotoxicity Assays

Cytotoxicities against human breast cancer (MDA-MB-231), colorectal cancer (HCT116), lung carcinoma (A549), pancreatic carcinoma (PANC-1), and hepatoma (HepG2) cell lines were evaluated using the SRB method (Skehan et al., 1990). Adriamycin was used as a positive control. The cell lines of MDA-MB-231, HCT116, A549, PANC-1, and HepG2 in the logarithmic growth phase were seeded into 96-well plates with 5,000 cells/well (100 μl/well), respectively. After 24 h of culture, the isolated compounds to be tested were added (the final concentration was shown in Supplementary Table 1), and three replicates were set for each concentration. The dosage of DMSO in the solvent control group was 0.1% of the maximum dose used in the test group. After 72 h of drug treatment, 10% (m/v) of cold trichloroacetic acid was added to each well to fix the cells. After SRB staining, 150 μl/well Tris solution was added to determine the optic density (OD) values at 515 nm on a microplate reader (TriStar^2^ S LB 942 Multimode Reader, Berthold Technologies, Germany). The inhibition rates of the tumor cell growth were calculated by the following formula:

$$\mathrm{Inhibition}\mathrm{rate}\left(\%\right)=\left({\text{OD}}_{\text{DMSO}}-{\mathrm{OD}}_{\text{compound}}\right)/{\text{OD}}_{\text{DMSO}}\times 100$$

The IC_50_ values were calculated using the method of log (inhibitor) vs. normalized response in the software package GraphPad Prism 5.

### Antibacterial Activity Assays

The antibacterial activities were evaluated by the conventional broth dilution assay (Appendino et al., 2008). Nine marine fouling bacteria, *Pseudomonas fulva*, *Aeromonas hydrophila*, *A. salmonicida*, *Vibrio anguillarum*, *V. harveyi*, *Photobacterium halotolerans*, *P. angustum*, *Enterobacter cloacae*, and *E. hormaechei*, were used, and cipofloxacin was used as a positive control. The initial screening of antibacterial activity assays was tested in a 96-well plate. Each well contained 198 μl tested bacterial suspension (2–5 × 10^5^ CFU/ml in LB broth) and 2 μl compound (final concentration was 20 μM). Three replicates were performed. The plates were incubated at 37°C for 24 h, and then the OD values were tested at 600 nm in a microplate reader (TriStar^2^ S LB 942 Multimode Reader, Berthold Technologies, Germany). The inhibitory rates were calculated according to the following formula:

$$\mathrm{Inhibition}\mathrm{rate}\left(\%\right)=\left({\text{OD}}_{\text{DMSO}}-{\mathrm{OD}}_{\text{compound}}\right)/{\text{OD}}_{\text{DMSO}}\times 100$$

The MIC values of some active target compounds were evaluated using the twofold serial dilution method. The concentrations of the compounds ranged from 100 to 6.25 μM. The other steps were the same as in the primary screening. The MIC values were calculated using the method of log (inhibitor) vs. normalized response in the software package GraphPad Prism 5.

## Results

### Structure Elucidations of Isolated Compounds

**FIGURE 3:**
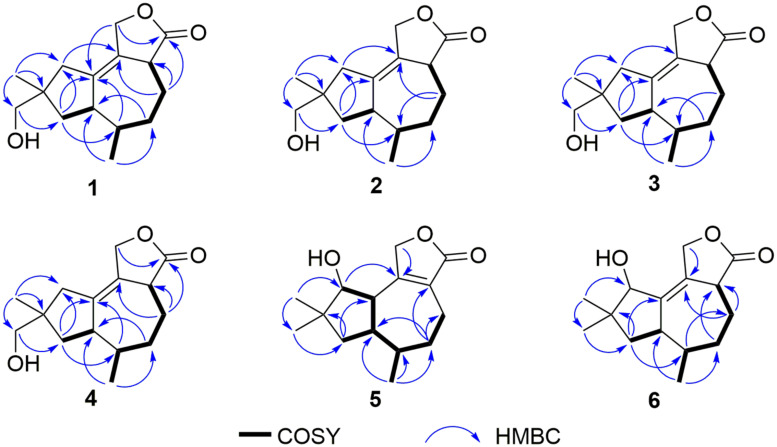
Key COSY and HMBC correlations of compounds **1**–**6**.

**FIGURE 4:**
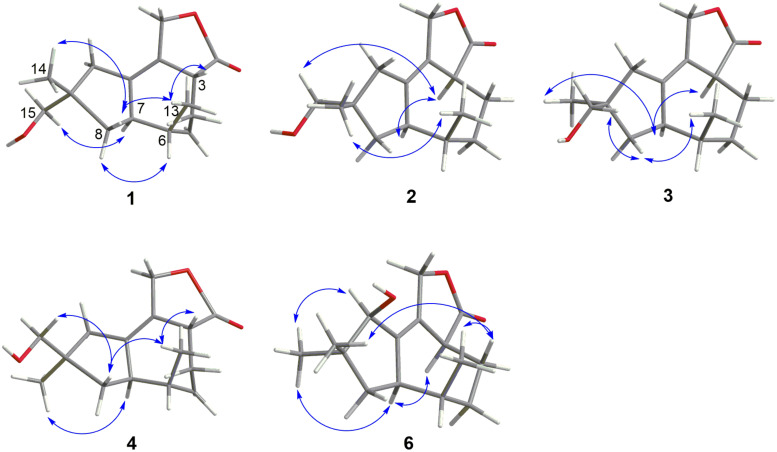
Key NOESY correlations of compounds **1**–**4** and **6**.

Pseudotremulane A (**1**) was obtained as a colorless oil. Its molecular formula, C_15_H_22_O_3_, was determined by the HR-APCI-MS spectrum (Supplementary Figure 7), with five degrees of unsaturation. The analysis of ^1^H NMR and ^13^C NMR spectra (Supplementary Figures 1, 2) combined with the HSQC spectrum (Supplementary Figure 3) of **1** indicated two methyl signals at δ_*H*_ 0.93 (3H, d, 7.2 Hz), δ_*C*_ 17.5; δ_*H*_ 1.11 (3H, s), δ_*C*_ 24.3, six methylenes, including two oxygenated methylenes at δ_*H*_ 3.39 (1H, d, 10.6 Hz), 3.46 (1H, d, 10.6 Hz), δ_*C*_ 69.18; δ_*H*_ 4.65 (1H, d, 10.3 Hz), 4.67 (1H, d, 10.3 Hz), δ_*C*_ 69.20, three methines, and four quaternary carbon signals, including two olefinic carbons at δ_*C*_ 125.0 and δ_*C*_ 138.5, and one ester group at δ_*C*_ 177.8, which represented two degrees of unsaturation (Tables 1, 2). The other degrees of unsaturation revealed that there had been three rings in the structure of **1**. These data suggested that **1** was tremulane-type sesquiterpenoid similar to 11,12-epoxy-12β-hydroxy-1-tremulen-5-one (**7**; Zhou et al., 2008). There had been three obvious differences between **1** and **7**. The disappeared ketone carbonyl in **7** was replaced by the arisen methylene at C-5 in **1** (Tables 1, 2); this was further confirmed by the key HMBC correlation from H-13 to C-5 (Figure 3). The HMBC correlations from H-11 to C-12, and H-4 to C-12 indicated the ester group carbon at C-12 (Figure 3). The lower field shift of C-15 data (Tables 1, 2) compared with those of **7**, combined with the HMBC correlations from H-15 to C-8, and H-14 to C-15 elucidated the oxidation of C-15 (Figure 3). Thus, the planer structure of **1** was unambiguously confirmed. The relative configurations of **1** were determined by NOESY spectra analysis (Supplementary Figure 6). The NOESY correlations between H-14 and H-8b, H-8b and H-13, and H-13 and H-3 indicated that H-14, H-13, and H-3 were in the same orientation (Figure 4). The other orientation of H-6, H-7, and H-15 was suggested by the NOESY cross-peaks of H-6/H-8a and H-7/H-15 (Figure 4). Therefore, the relative configurations of **1** were assigned as 3*R*^∗^,6*R*^∗^,7*R*^∗^,9*S*^∗^.

Pseudotremulane B (**2**) was gained as a colorless oil, with the molecular formula of C_15_H_22_O_3_ determined by HR-APCI-MS indicating five degrees of unsaturation and had the same molecular formula as **1** (Supplementary Figure 14). The ^1^H and ^13^C NMR data of **2** were very similar to those of **1** (Tables 1, 2). The downfield shift of C-2, C-3, C-5, C-7, C-9, C-11, and C-12 and the high-field shift of C-4, C-6, C-8, C-13, and C-14 in ^13^C NMR suggested the difference configurations between **1** and **2**. The NOESY cross-peaks of H-15/H-3 and H-3/H-7 declared that H-3, H-7, and H-15 were in the same face (Figure 4). The NOESY correlation of H-13 and H-14 indicated that H-13 and H-14 were in another face. Therefore, the relative configurations of **2** were assigned as 3*S*^∗^,6*R*^∗^,7*R*^∗^,9*S*^∗^.

Pseudotremulane C (**3**) was acquired as a colorless oil. The HR-APCI-MS of **3** exhibited the same molecular formula with **1** and **2** (Supplementary Figure 21). The strong similar ^1^H and ^13^C NMR data between **2** and **3** (Tables 1, 2) suggested that they shared the same planer structures. The high-field shift of C-14 and the downfield shift of C-15 (Table 2) revealed the difference configurations of C-9 of **2** and **3**. The α-orientation of H-3, H-7, and H-14 was determined by the NOESY correlations of H-3/H-7 and H-7/H-14 (Figure 4). The β-orientation of H-13 and H-15 was determined by the NOESY cross-peaks of H-13/H-8a and H-8a/H-15 (Figure 4). Compounds **2** and **3** were a pair of epimeride at the location of C-9.

Pseudotremulane D (**4**) was obtained as a colorless oil, with the same molecular formula with **1**–**3**, according the analysis of its HR-APCI-MS spectrum (Supplementary Figure 28). Careful analysis of the ^1^H and ^13^C NMR data of **1** and **4** indicated that they had the same planer structures. The difference configurations of C-9 of **1** and **4** were determined by the high-field shift of C-14 and the downfield shift of C-15 (Table 2). The NOESY correlations of H-3/H-13, H-13/H-8a, and H-8a/H-15 (Figure 4) revealed the β-orientation of H-3, H-13, and H-15. The α-orientation of H-7 and H-14 was proved by the NOESY cross-peak of H-7/H-14 (Figure 4). Compounds **1** and **4** were a pair of epimeride at the location of C-9.

Pseudotremulane E (**5**) was obtained as a colorless oil. Its molecular formula was the same as **1**–**4**, as suggested by HR-APCI-MS (Supplementary Figure 35). The NMR spectra of **5** revealed the presence of three methyls, four methylenes (one oxygenated), four methines (one oxygenated), and four quaternary carbons (one ester group carbon, two olefinic, and one sp^3^ quaternary carbon; Supplementary Figures 29–31). These characteristic NMR spectroscopic data of **5** showed similarities with those of 11,12-epoxy-12β-hydroxy-1-tremulen-5-one (**7**; Zhou et al., 2008). Compared with **7**, the disappeared ketone at C-5 was substituted by methylene [δ_*H*_ 1.71–1.62 (2H, m), δ_*C*_ 33.5] in **5** (Tables 1, 2), elucidated by the ^1^H-^1^H COSY correlations of H-6/H-5 and H-5/H-4, and further confirmed by the HMBC correlations from H-13 to C-5, and H-5 to C-3, C-4, and C-7 (Figure 3). The position of the double bond was changed from C-1/C-2 in **7** into C-2/C-3 in **5**, proved by the ^1^H-^1^H COSY cross-peak of H-1/H-7 and the HMBC signals of H-10/C-2, H-11/C-2, and H-5/C-3 (Figure 3). The absence of carbonyl carbon (δ_*C*_ 175.3) in **5** and the disappeared oxygenated methine at C-12 in **7**, combined with the molecular formula of **5**, revealed that there had been an ester group at C-12 in **5**. The large coupling constants of H-1/H-10 (*J* = 11.0 Hz) and H-1/H-7 (*J* = 11.0 Hz) revealed the β-orientation of H-1 and the α-orientation of H-7 and H-10 (Table 1). The overlapped ^1^H NMR signals of H-6/H-7 and H-13/H-14 increased the difficulties to decide the configurations of **5** (Table 1). However, based on biogenetic considerations, H-13 was proposed to have β-orientation be the same with **1**–**6.**

Pseudotremulane F (**6**) was isolated as a colorless oil. The same molecular formula of C_15_H_22_O_3_ was determined by the HR-APCI-MS spectrum (Supplementary Figure 42). The three methyls, four methylenes (one oxygenated), four methines (one oxygenated), and four quaternary carbons (one ester group carbon, two olefinic, and one sp^3^ quaternary carbon) exhibited in the NMR spectra (Supplementary Figures 29–31), indicating the similar structures of **6** and **5**. The most obvious differences of ^13^C NMR data between **6** and **5** were the downfield shift of C-1 (δ_*C*_ 140.8 in **6** *vs* δ_*C*_ 45.0 in **5**) and the high-field shift of C-2 (δ_*C*_ 132.6 in **6** *vs* δ_*C*_ 162.9 in **5**) and C-3 (δ_*C*_ 44.7 in **6** *vs* δ_*C*_ 128.8 in **5**; Table 2), elucidating that the olefinic bond location was changed from C-2/C-3 in **5** into C-1/C-2 in **6**. This was further confirmed by the HMBC correlations from H-11 to C-2, H-4 to C-2, and H-8 to C-1 (Figure 3). The β-orientation of H-13 and H-14 was revealed by the NOESY cross-peaks of H-13/H-4b and H-4b/H-14, and the α-orientation of H-3, H-7, H-15, and H-10 was suggested by the NOESY correlations of H-3/H-7, H-7/H-15, and H-15/H-10 (Figure 4).

**FIGURE 5:**
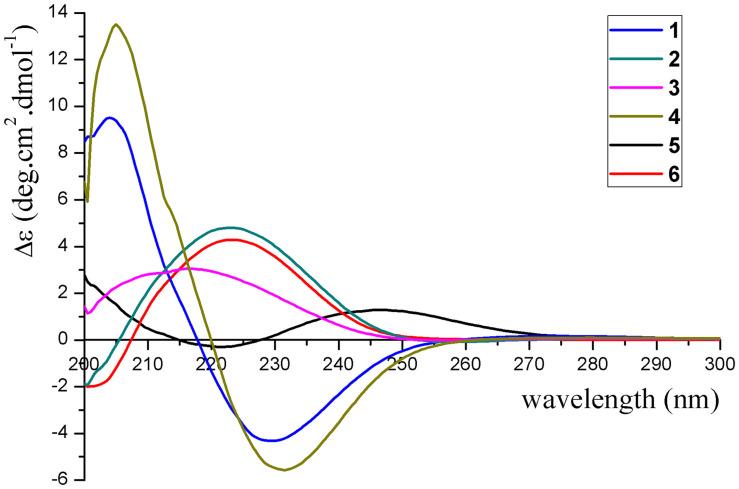
Experimental ECD spectra of compounds **1**–**6**.

The absolute configurations of **1**–**6** were determined by their ECD spectra (Figure 5) and were further confirmed by ECD calculations. The experimental ECD spectrum of **1** exhibited a negative cotton effect at 230 nm. According to the π-π^∗^ CD octant rule for olefins (Guo et al., 2016), the negative cotton effect at 230 nm was caused by ester carbonyl (C-12) and oxymethene (C-11) lying in the negative contribution region (Figure 5). Combined with the relative configuration conclusions, the absolute configurations of **1** were established as 3*R*,6*R*,7*R*,9*S*, and named as pseudotremulane A. The similar ECD spectra of **4** and **5** with the negative cotton effects at 232 and 222 nm (Figure 5), respectively, indicated the absolute configurations of 3*R*,6*R*,7*R*,9*R*–**4** and 1*S*,6*R*,7*R*,10*S*–**5**. The positive cotton effects of the ECD spectra of compounds **2** (223 nm), **3** (217 nm), and **6** (223 nm) elucidated the absolute configurations of 3*S*,6*R*,7*R*,9*S*–**2**, 3*S*,6*R*,7*R*,9*R*–**3**, and 3*S*,6*R*,7*R*,10*R*–**6** (Figure 5). Thus, the structures compounds **2**–**6** were completely confirmed and named as pseudotremulanes B-F, respectively.

**FIGURE 6:**
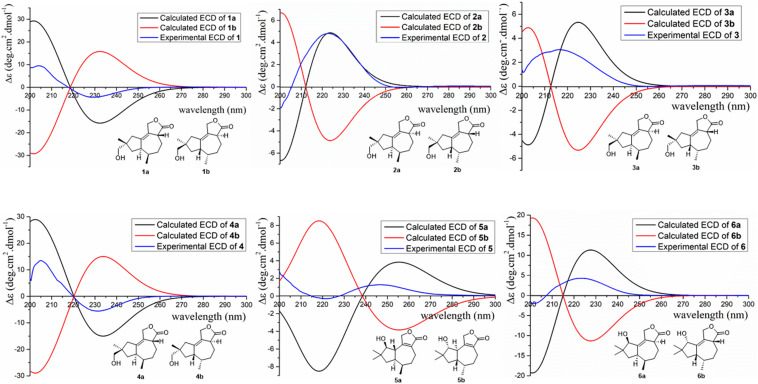
Experimental and calculated ECD of compounds **1**–**6**.

To further conform these results, the theoretical ECDs of compounds **1**–**6** (Figure 6) were calculated to compare with their experimental ECD spectra (Mazzeo et al., 2013; Cao et al., 2020). The MMFF94S method was used to conformational searches of **1a**–**6a** to obtain the lowest energy conformers with relative energies between 0 and 10 kcal/mol. Gaussian 09 package was used to optimize the searched conformations. The first optimization was set at the gas-phase RB3LYP/6-31G(d) level to get preferential conformations with the relative energies less than 2.5 kcal/mol. Then the conformers were optimized again at the set of gas-phase B3LYP/6-311 + G(d). The total 60 electronic excited states were calculated at the set of gas-phase RB3LYP/6-311 + + G(2d,p). Boltzmann statistics were used to simulate ECD with a standard deviation of σ 0.4 eV. The theoretical ECD spectra of **1b**–**6b** were obtained by directly reversing the spectra of **1a**–**6a**, respectively. The results exhibited that the experimental ECDs of **1**–**6** were matched well with the calculated ECDs of **1a**–**6a**, respectively, which further verified the absolute structures of **1**–**6** (Figure 6). Interestingly, compounds **1**/**2**, **3**/**4**, **1**/**4**, and **2**/**3** were identified as four pairs of epimeride at the locations of C-3, C-3, C-9, and C-9, respectively.

The structures of **7**–**12** were determined as 11,12-epoxy-12β-hydroxy-1-tremulen-5-one (Zhou et al., 2008), ganodermasides A, B, and D (Weng et al., 2010, 2011), ergosterol (Feng et al., 2010), and dankasterone B (Amagata et al., 2007), respectively, by comparing their NMR data with those in the literature.

### Proposed Biosynthetic Pathway for 1–7

**FIGURE 7:**
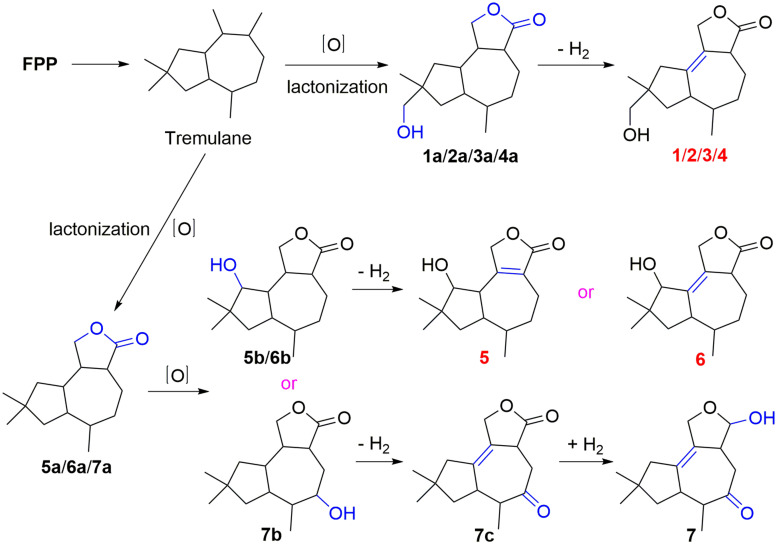
Proposed biosynthetic pathway for **1**–**7**.

Compounds **1**–**7** could derive from tremulane, a 5/7 endocyclic system sesquiterpenoid (Figure 7; He et al., 2020; Wang et al., 2020). As exhibited in Figure 7, compounds **1**–**7** could be obtained after a series of oxidation, lactonization, dehydrogenation, and revivification of tremulane. The intermediate products **1a**/**2a**/**3a**/**4a** were obtained after the oxidation of tremulane at C-11, C-12, and C-15 and lactonization at C-11 and C-12. Then the dehydrogenation of the intermediate products at C-1 and C-2 acquired the compounds **1**–**4**. Similarly, compounds **5**, **6**, and **7c** were gained from tremulane after the reactions of oxidation, lactonization, and dehydrogenation. Compound **7** was obtained from the revivification of **7c**.

### Bioactivity Evaluations of Isolated Compounds

**FIGURE 8:**
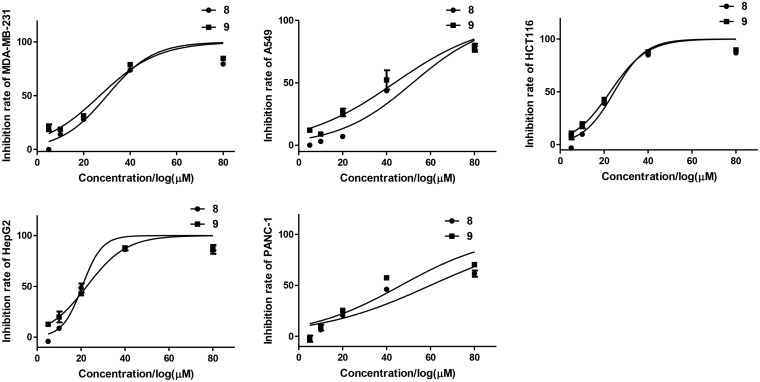
Inhibition rates of tested cell lines of compounds **8** and **9**.

All the isolated compounds (**1**–**12**) were evaluated for their cytotoxic activities against five human cancer cell lines (MDA-MB-231, HCT116, HepG2, A549, and PANC-1). Compounds **8** and **9** exhibited cytotoxicities against MDA-MB-231, HCT116, and HepG2 cell lines with the IC_50_ values ranging from 21 to 30 μM (Table 3 and Figure 8).

**TABLE 3:** Cytotoxicities (IC_50_, μM) of compounds **8** and **9**.

**Compounds**	**8**	**9**
MDA-MB-231	30 ± 2.0	27 ± 1.7
A549	>40	>40
HCT116	25 ± 1.5	23 ± 0.93
HepG2	21 ± 1.0	23 ± 1.3
PANC-1	>40	>40

**FIGURE 9:**
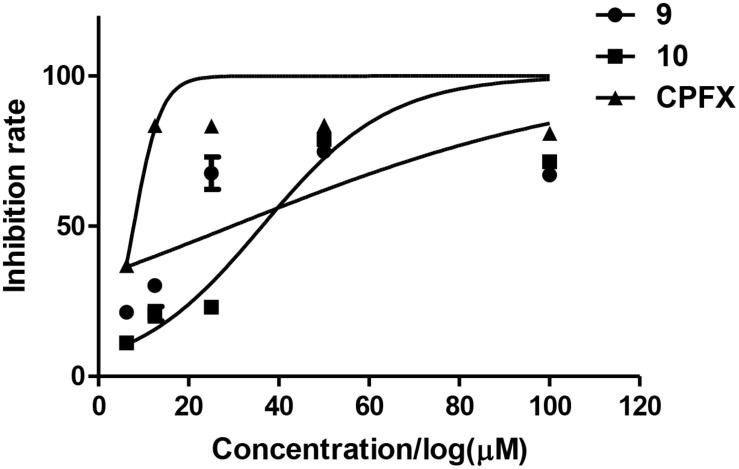
Inhibition rates of *A. salmonicida* of compounds **9** and **10**.

The antibacterial activities of the isolated compounds (**1**–**12**) were also evaluated against nine marine fouling bacteria *P. fulva*, *A. hydrophila*, *A. salmonicida*, *V. anguillarum*, *V. harveyi*, *P. halotolerans*, *P. angustum*, *E. cloacae*, and *E. hormaechei*. Compounds **9** and **10** showed antibacterial activities against marine fouling bacteria *A. salmonicida* with the MIC values of 30 and 36 μM, respectively (Figure 9). The MIC value of the positive control ciprofloxacin (CPFX) was 7.8 μM (Figure 9).

## Conclusion

In summary, six new tremulane sesquiterpenoids, pseudotremulanes A–F (**1**–**6**), together with one known analog, 11,12-epoxy-12β-hydroxy-1-tremulen-5-one (**7**), and five known steroids, ganodermasides A (**8**), B (**9**), and D (**10**), ergosterol (**11**), and dankasterone B (**12**), were isolated from the Antarctic-derived fungus *Pseudogymnoascus* sp. HSX2#-11. Compounds **1**–**7** were proved to be isomeride structures with the same chemical formula. Compounds **1** and **2**, **3** and **4**, **1** and **4**, and **2** and **3** were identified as four pairs of epimeride at the locations of C-3, C-3, C-9, and C-9, respectively. Compounds **8** and **9** exhibited cytotoxicities against MDA-MB-231, HCT116, and HepG2 cell lines. Compounds **9** and **10** showed antibacterial activities against marine fouling bacteria *A. salmonicida*. This is the first time to discover terpenoids and steroids from the fungal genus *Pseudogymnoascus*. Our chemical investigation of the Antarctic fungus *Pseudogymnoascus* sp. HSX2#-11 enriches the chemical diversity of this fungal species.

## Discussion

The genus *Pseudogymnoascus* as a kind of psychrophilic pathogenic fungi is widely distributed in Antarctica (Rosa et al., 2020; Santos et al., 2020; Martorell et al., 2021). *Pseudogymnoascus* can be one of the antagonistic fungi against potato scab pathogens from potato field soils, which could be used as potential agents to control potato scab disease (Tagawa et al., 2010). *Pseudogymnoascus* spp. has been certified to be one of the predominant microbial colonizers in the root endosphere and rhizosphere of turfgrass systems (Xia et al., 2021). The extracts of some *Pseudogymnoascus* strains exhibit potent bioactivities, such as antimicrobial, herbicidal, and antitumoral activities (Henríquez et al., 2014; Gonçalves et al., 2015; Gomes et al., 2018; Ferrarezi et al., 2019). To the best of our knowledge, only 22 natural products, including 6 new compounds, were discovered from *Pseudogymnoascus* up to now (Figueroa et al., 2015; Guo et al., 2019; Fujita et al., 2021; Shi et al., 2021). More than 70% of the previously isolated structures belong to polyketides; others are alkaloids (13.6%), benzene derivative (9.1%), and fatty acid (4.5%). Our research isolated 12 natural products (**1**–**12**), including 6 new compounds (**1**–**6**), from the fungus *Pseudogymnoascus* 2#-11. All of the isolated compounds are first obtained from the genus *Pseudogymnoascus*. This is the first time to discover terpenoids and steroids from the genus *Pseudogymnoascus*. The whole number of the fungal strain secondary metabolites increased by 35%, and the number of their new compounds is doubled. This greatly enriches the number and diversity of natural products of the genus *Pseudogymnoascus*. Except for antimicrobial activities of some of the previously obtained polyketides (Figueroa et al., 2015; Fujita et al., 2021; Shi et al., 2021), no other activities were found in *Pseudogymnoascus* in previous studies. This study is the first to identify secondary metabolites with cytotoxic activities (**8** and **9**) in *Pseudogymnoascus*.

The isolated new sesquiterpenoids (**1**–**6**), with characteristic structures of 5/7 fused bicyclic system, belong to the family of tremulanes. Tremulane derivatives have been found from cultures of *Phellinus tremulae* (Ayer and Cruz, 1993), *P. igniarius* (Liu et al., 2007; Wu et al., 2020), *Conocybe siliginea* (Zhou et al., 2008; Wu et al., 2010; He et al., 2020), *Huperzia serrata* (Ying et al., 2013), *Flavodon flavus* (Isaka et al., 2016), *Coriolopsis* sp. (Chen et al., 2017), *Colletotrichum capsici* (Wang et al., 2017), *I. lacteus* (Chen et al., 2018, 2020; Ding et al., 2018, 2019, 2020a,b; Zhou et al., 2018; Duan et al., 2019; Wu et al., 2019; Shi et al., 2020; Sun C.-T. et al., 2020; Wang et al., 2020), and *Gymnopilus junonius* (Lee et al., 2020). This is the first time to find tremulane derivatives from *Pseudogymnoascus*.

## Data Availability Statement

The datasets presented in this study can be found in online repositories. The names of the repository/repositories and accession number(s) can be found in the article/Supplementary Material.

## Author Contributions

TS contributed to experimental design and operation, data analysis, and manuscript preparation. X-QL contributed to manuscript revision. LZ supported the sample of the Antarctic soil. Y-HZ contributed to ECD calculations. J-JD contributed to activity evaluations. E-LS contributed to software drawing guidance. Y-YY, Y-TZ, and W-PH contributed to activity evaluations. D-YS was the project leader organizing and guiding the experiments. All authors contributed to the article and approved the submitted version.

## Conflict of Interest

The authors declare that the research was conducted in the absence of any commercial or financial relationships that could be construed as a potential conflict of interest.
