# Supplementary material for: Sesquiterpenoids From the Antarctic Fungus Pseudogymnoascus sp. HSX2#-11
Source: Front Microbiol. 2021 Jun 11;12:688202. doi: 10.3389/fmicb.2021.688202 (PMC8226235; doi:10.3389/fmicb.2021.688202)
Supplement: Supplementary file 1 [file Data_Sheet_1.PDF]

# *Supplementary Material*

# Sesquiterpenoids from the Antarctica Fungus *Pseudogymnoascus* sp. HSX2#-11

Ting Shi[1], Xiang-Qian Li[1,2], Li Zheng[3.4], Ya-Hui Zhang[5.6], Jia-Jia Dai[1], Er-Lei Shang[7], Yan-Yan Yu[1], Yi-Ting Zhang[1], Wen-Peng Hu[1], and Da-Yong Shi[1,2*]

[1]State Key Laboratory of Microbial Technology, Institute of Microbial Technology, Shandong University, Qingdao 266200, China.

[2]Laboratory for Marine Drugs and Bioproducts of Qingdao National Laboratory for Marine Science and Technology, Qingdao 266071, China.

[3]Key Laboratory of Marine Eco-Environmental Science and Technology, First Institute of Oceanography, Ministry of Natural Resources, Qingdao, 266061, China.

[4]Laboratory for Marine Ecology and Environmental Science, Qingdao Pilot National Laboratory for Marine Science and Technology, Qingdao, 266071, China.

[5]Key Laboratory of Marine Drugs, The Ministry of Education of China, School of Medicine and Pharmacy, Ocean University of China, Qingdao 266003, China.

[6]Laboratory for Marine Drugs and Bioproducts, Qingdao National Laboratory for Marine Science and Technology, Qingdao 266071, China.

[7]State Key Laboratory of Pharmaceutical Biotechnology, School of Life Sciences, Nanjing University, Nanjing 210023, China

**Figure S1.** $^{1}H$ NMR (600 MHz, $CDCl_3$) spectrum of compound **1**
**Figure S2.** $^{13}C$ NMR (150 MHz, $CDCl_3$) spectrum of compound **1**
**Figure S3.** HSQC ($CDCl_3$) spectrum of compound **1**
**Figure S4.** COSY ($CDCl_3$) spectrum of compound **1**
**Figure S5.** HMBC ($CDCl_3$) spectrum of compound **1**
**Figure S6.** NOESY ($CDCl_3$) spectrum of compound **1**
**Figure S7.** HR-APCI-MS spectrum of compound **1**
**Figure S8.** $^{1}H$ NMR (600 MHz, $CDCl_3$) spectrum of compound **2**
**Figure S9.** $^{13}C$ NMR (150 MHz, $CDCl_3$) spectrum of compound **2**
**Figure S10.** HSQC ($CDCl_3$) spectrum of compound **2**
**Figure S11.** COSY ($CDCl_3$) spectrum of compound **2**
**Figure S12.** HMBC ($CDCl_3$) spectrum of compound **2**
**Figure S13.** NOESY ($CDCl_3$) spectrum of compound **2**
**Figure S14.** HR-APCI-MS spectrum of compound **2**
**Figure S15.** $^{1}H$ NMR (600 MHz, $CDCl_3$) spectrum of compound **3**
**Figure S16.** $^{13}C$ NMR (150 MHz, $CDCl_3$) spectrum of compound **3**
**Figure S17.** HSQC ($CDCl_3$) spectrum of compound **3**
**Figure S18.** COSY ($CDCl_3$) spectrum of compound **3**
**Figure S19.** HMBC ($CDCl_3$) spectrum of compound **3**
**Figure S20.** NOESY ($CDCl_3$) spectrum of compound **3**
**Figure S21.** HR-APCI-MS spectrum of compound **3**
**Figure S22.** $^{1}H$ NMR (600 MHz, $CDCl_3$) spectrum of compound **4**
**Figure S23.** $^{13}C$ NMR (150 MHz, $CDCl_3$) spectrum of compound **4**
**Figure S24.** HSQC ($CDCl_3$) spectrum of compound **4**
**Figure S25.** COSY ($CDCl_3$) spectrum of compound **4**
**Figure S26.** HMBC ($CDCl_3$) spectrum of compound **4**
**Figure S27.** NOESY ($CDCl_3$) spectrum of compound **4**
**Figure S28.** HR-APCI-MS spectrum of compound **4**
**Figure S29.** $^{1}H$ NMR (600 MHz, $CDCl_3$) spectrum of compound **5**
**Figure S30.** $^{13}C$ NMR (150 MHz, $CDCl_3$) spectrum of compound **5**
**Figure S31.** HSQC ($CDCl_3$) spectrum of compound **5**
**Figure S32.** COSY ($CDCl_3$) spectrum of compound **5**
**Figure S33.** HMBC ($CDCl_3$) spectrum of compound **5**
**Figure S34.** NOESY ($CDCl_3$) spectrum of compound **5**
**Figure S35.** HR-APCI-MS spectrum of compound **5**
**Figure S36.** $^{1}H$ NMR (600 MHz, $CDCl_3$) spectrum of compound **6**
**Figure S37.** $^{13}C$ NMR (150 MHz, $CDCl_3$) spectrum of compound **6**
**Figure S38.** HSQC ($CDCl_3$) spectrum of compound **6**
**Figure S39.** COSY ($CDCl_3$) spectrum of compound **6**

**Figure S40.** HMBC ($CDCl_3$) spectrum of compound **6**
**Figure S41.** NOESY ($CDCl_3$) spectrum of compound **6**
**Figure S42.** HR-APCI-MS spectrum of compound **6**
**Table S1.** Inhibition rates of tested cell lines of compounds **8** and **9**.
**Table S2.** Inhibition rates of tested marine fouling bacteria of compounds **1−12**.
**Table S3.** Inhibition rates of *A. salmonicida* of compounds **9** and **10**.

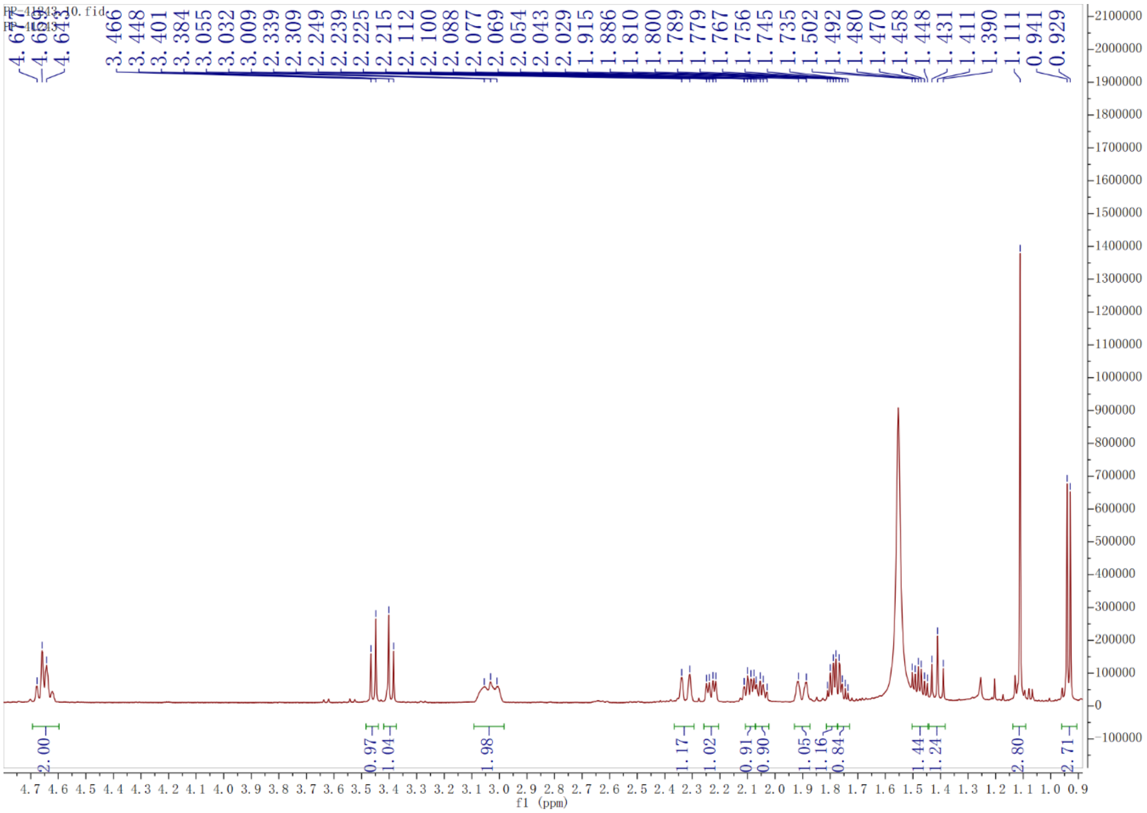

**Figure S1.** $^{1}H$ NMR (600 MHz, $CDCl_3$) spectrum of compound **1**

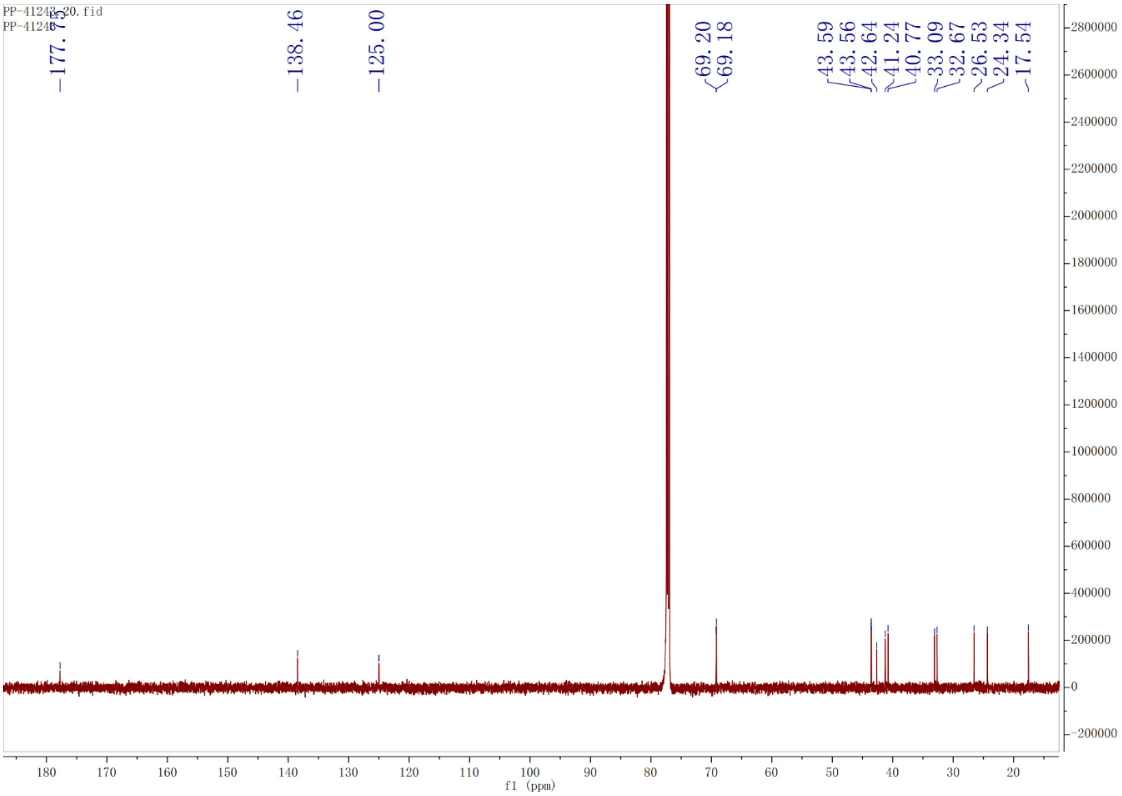

**Figure S2.** $^{13}C$ NMR (150 MHz, $CDCl_3$) spectrum of compound **1**

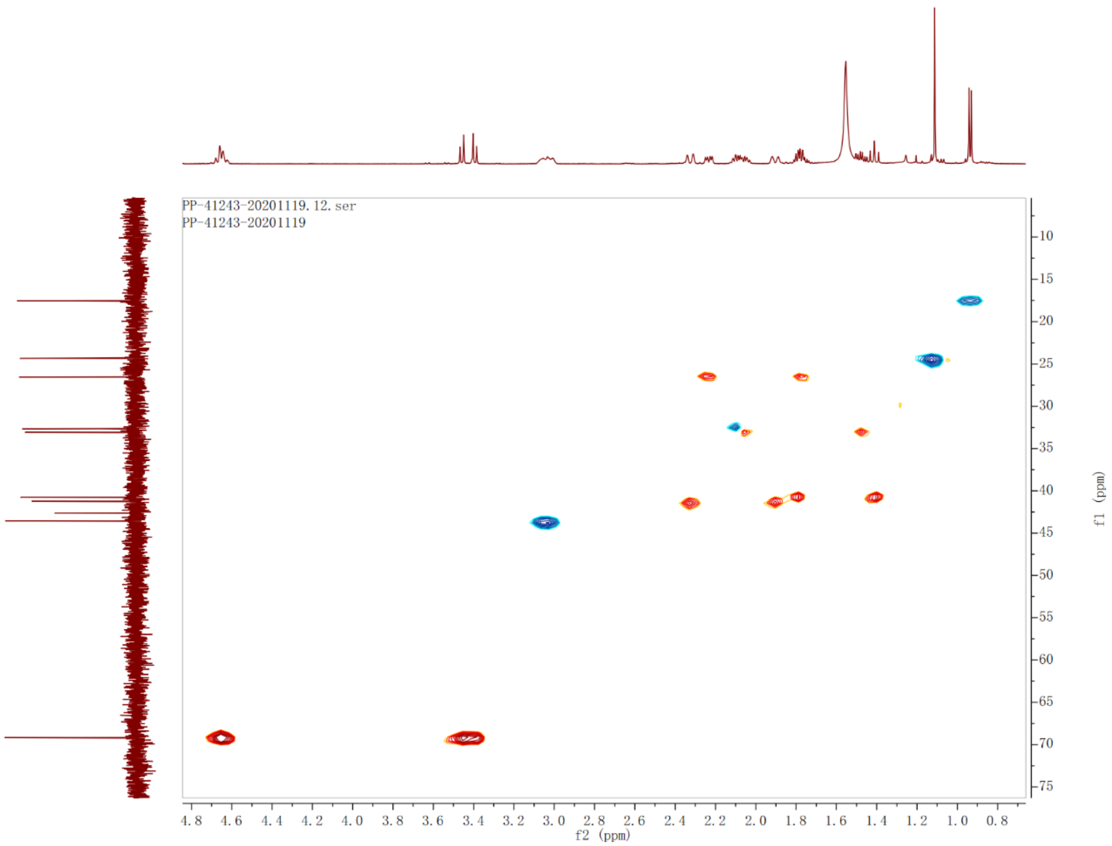


**Figure S3.** HSQC ($CDCl_3$) spectrum of compound **1**

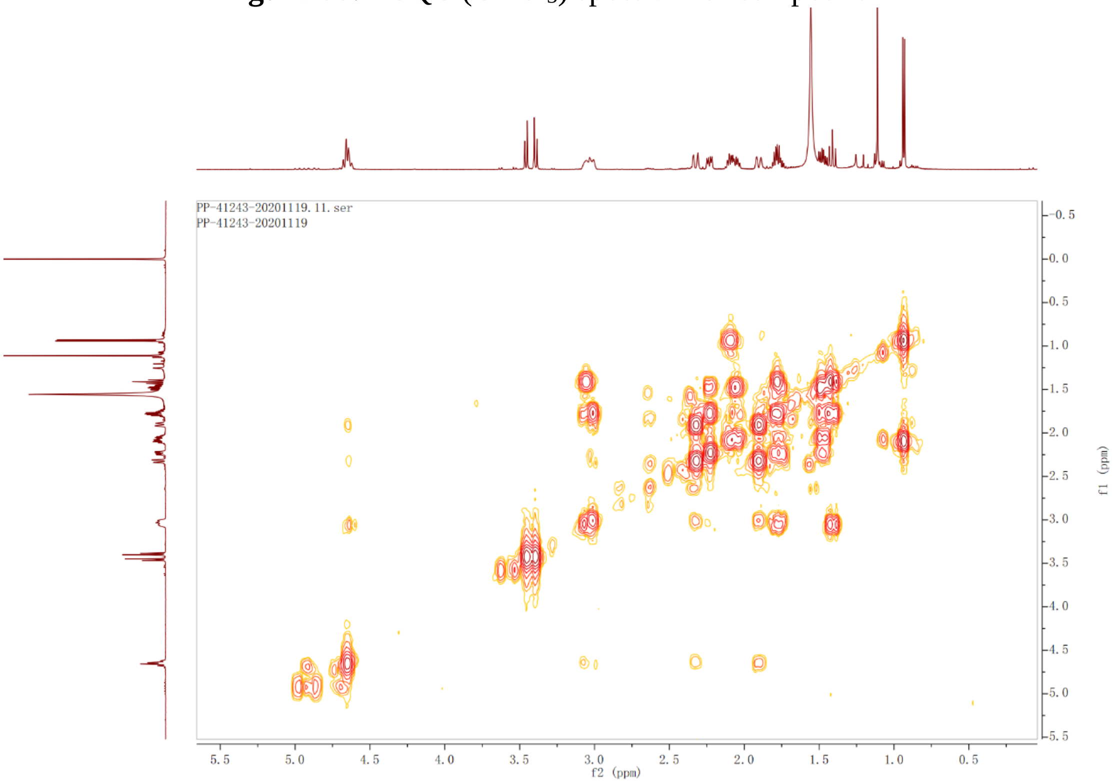


**Figure S4.** COSY ($CDCl_3$) spectrum of compound **1**

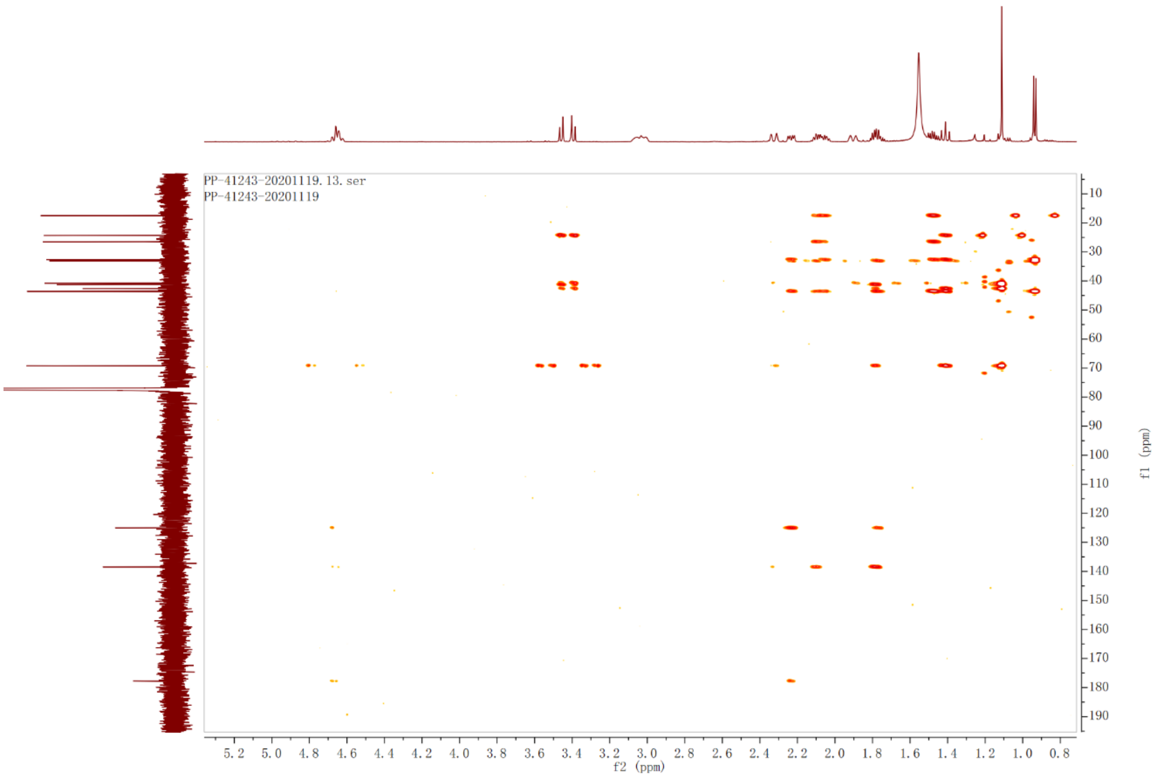


**Figure S5.** HMBC ($CDCl_3$) spectrum of compound **1**

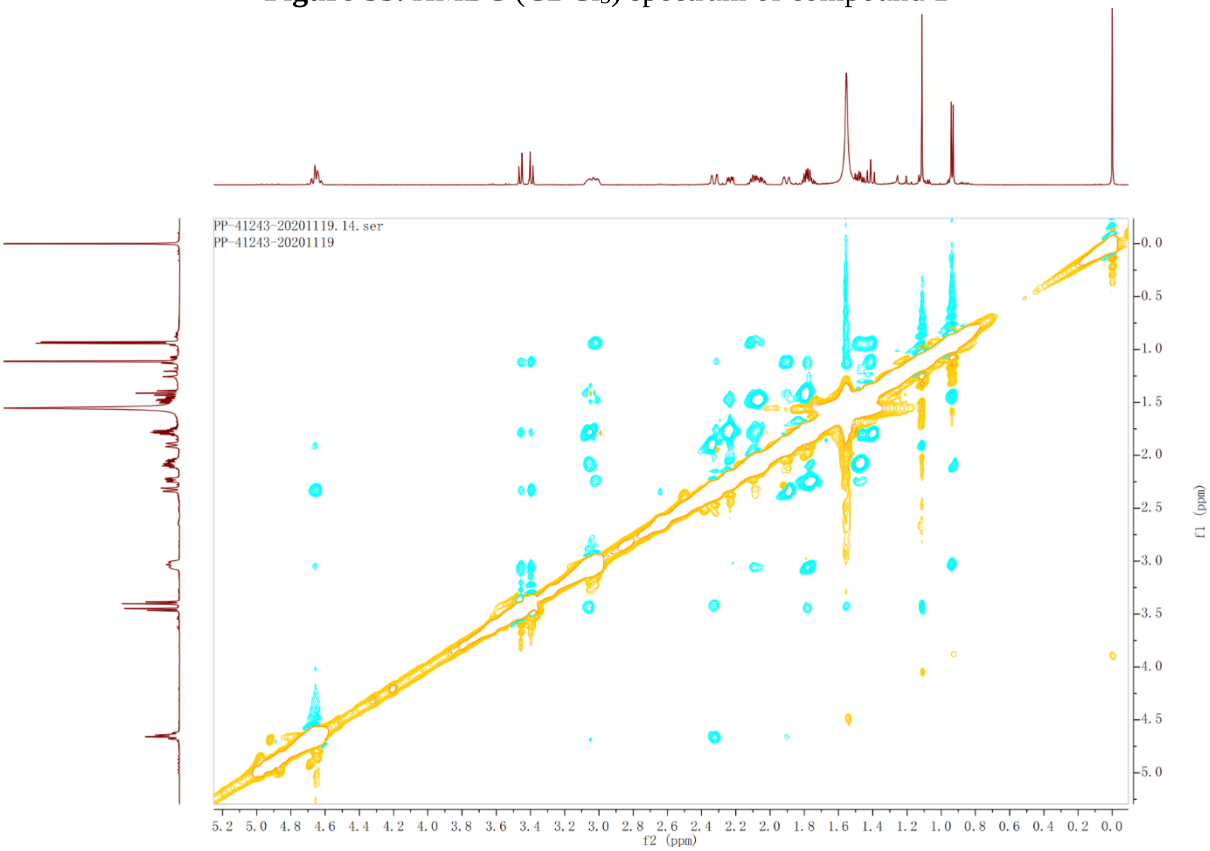


**Figure S6.** NOESY ($CDCl_3$) spectrum of compound **1**

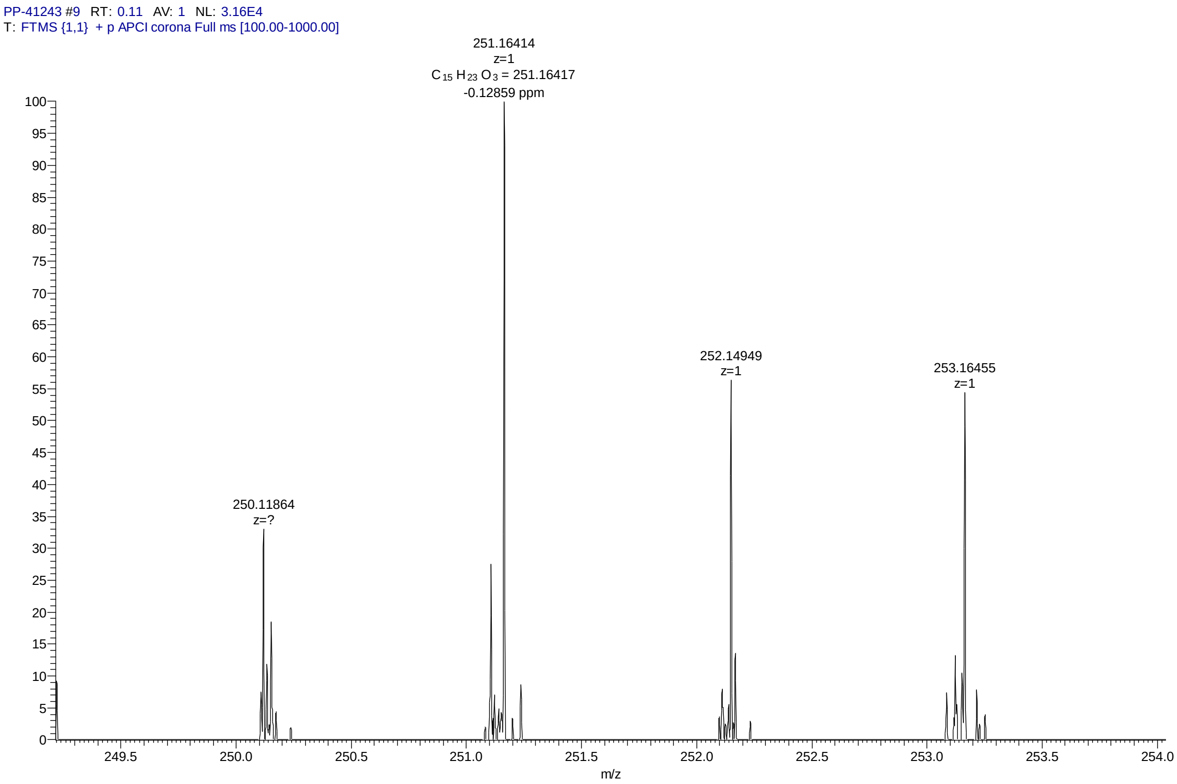


**Figure S7.** HR-APCI-MS spectrum of compound **1**

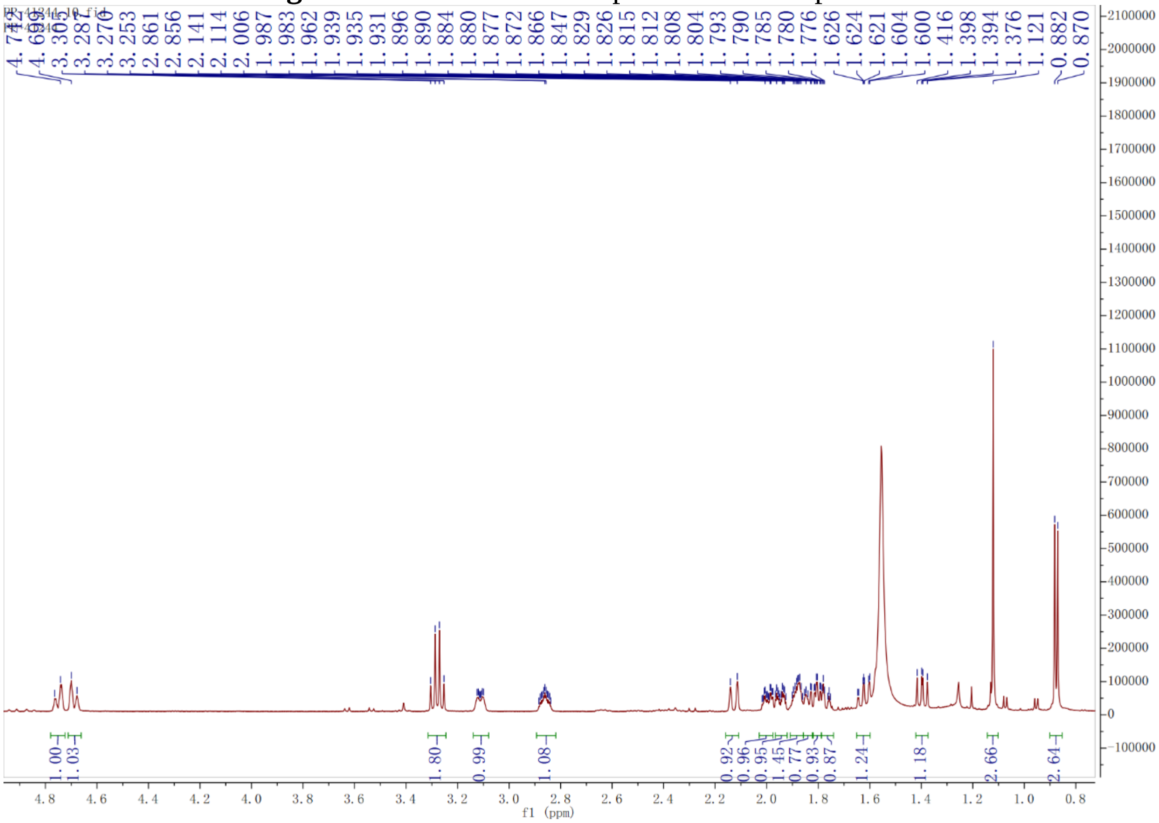


**Figure S8.** $^{1}$H NMR (600 MHz, $CDCl_3$) spectrum of compound **2**

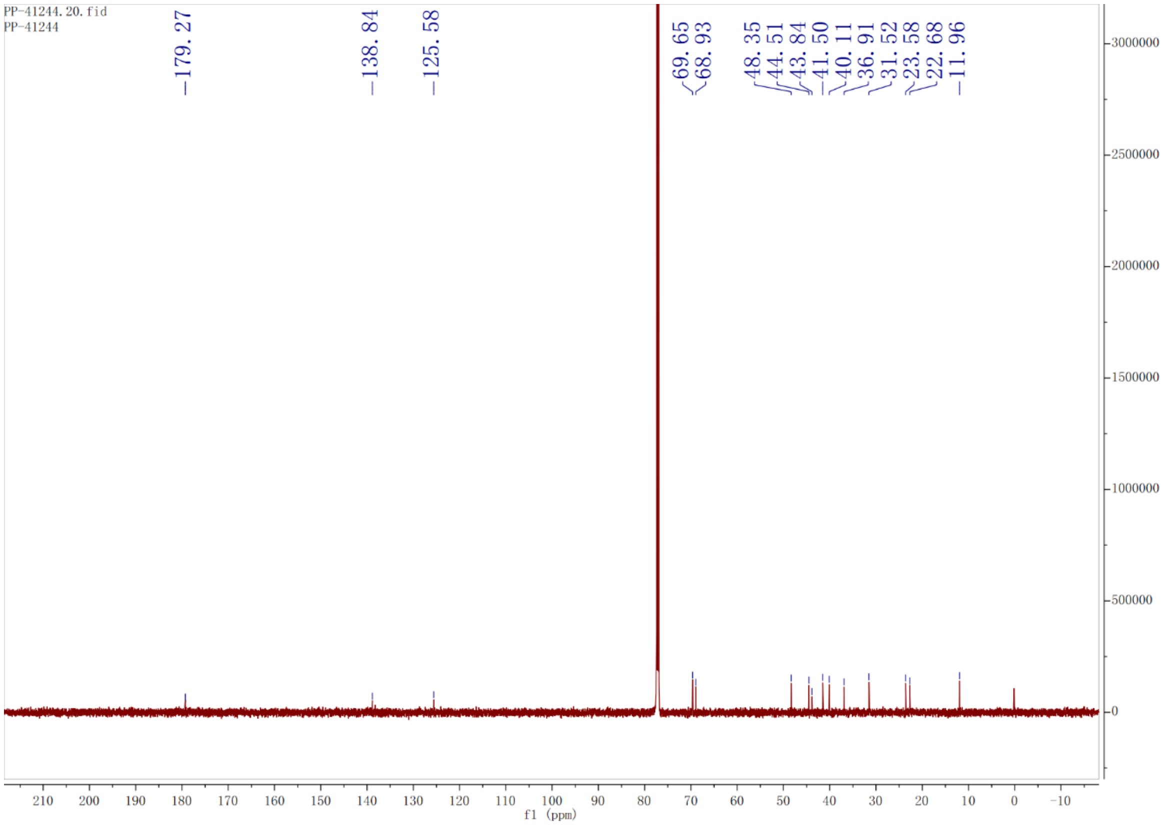


**Figure S9.** $^{13}$C NMR (150 MHz, $CDCl_3$) spectrum of compound **2**

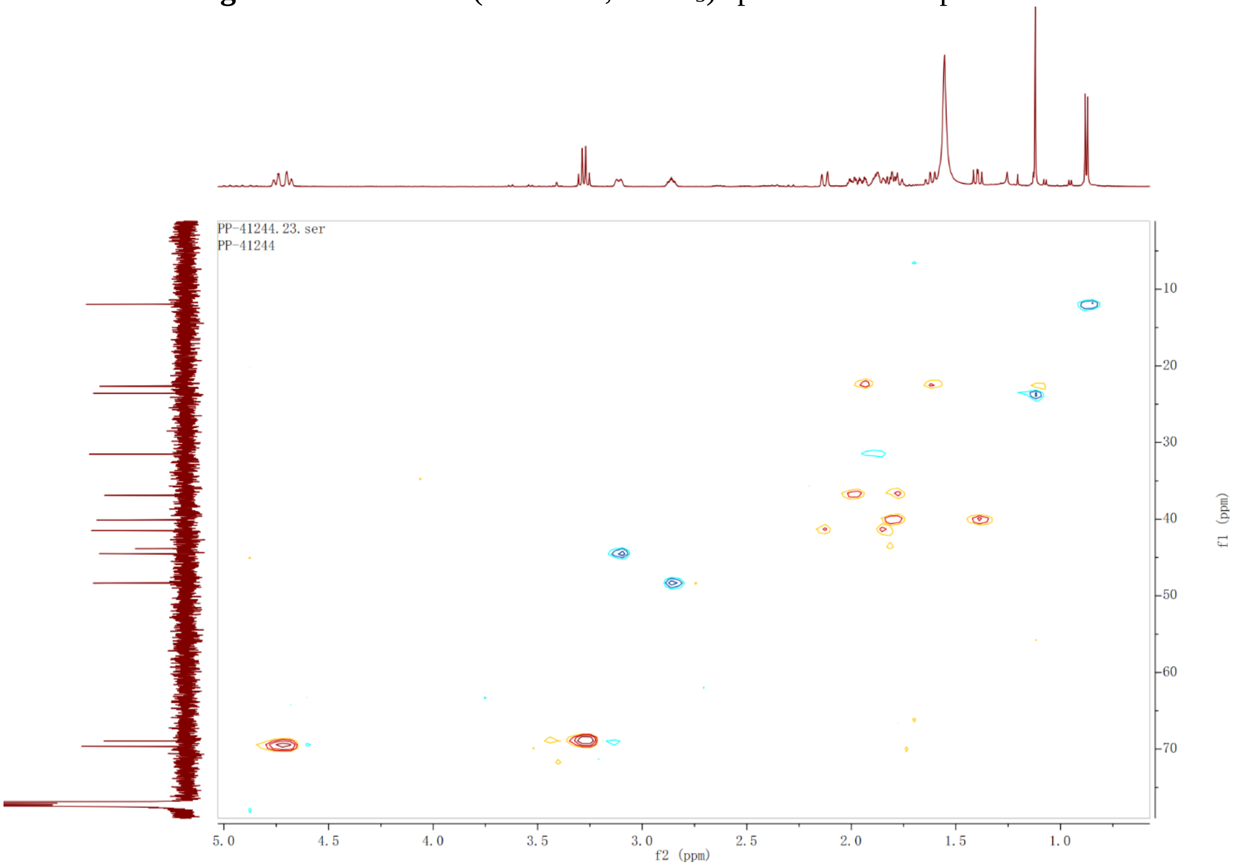


**Figure S10.** HSQC ($CDCl_3$) spectrum of compound **2**

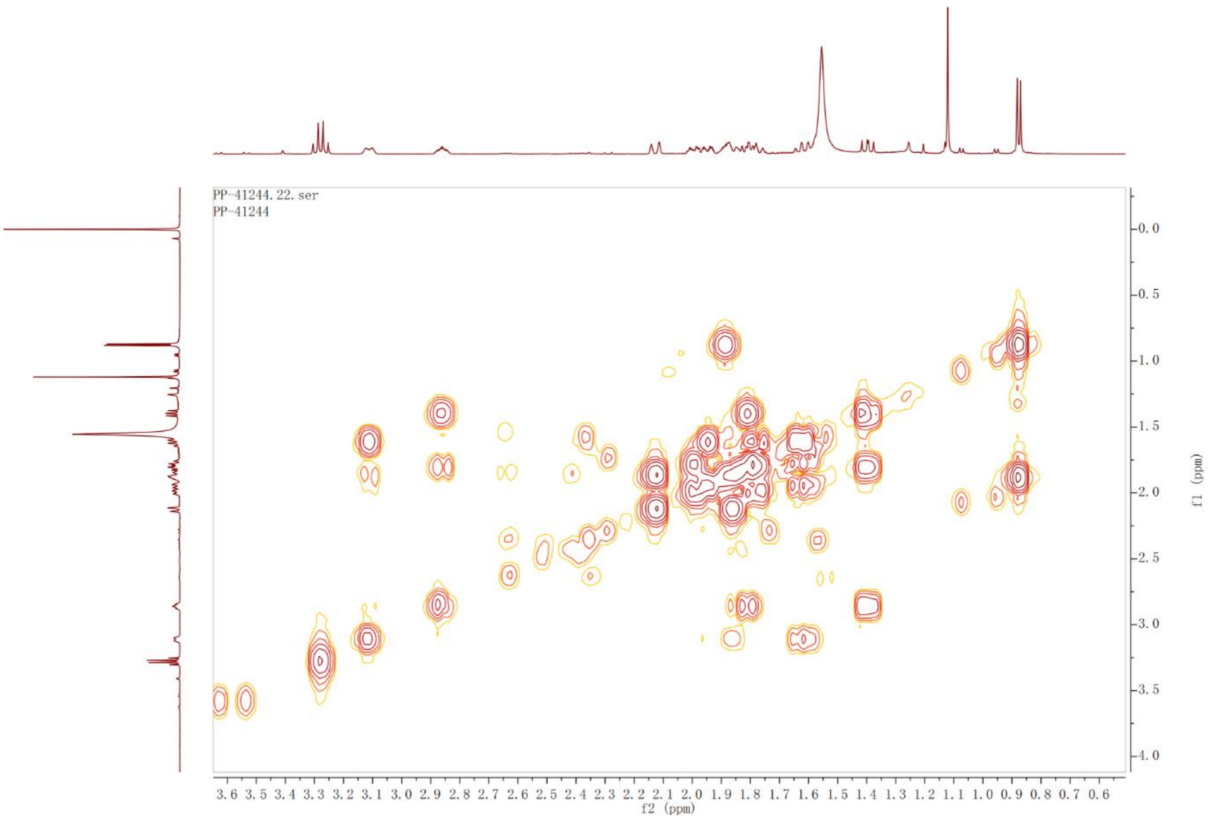


**Figure S11.** COSY ($CDCl_3$) spectrum of compound **2**

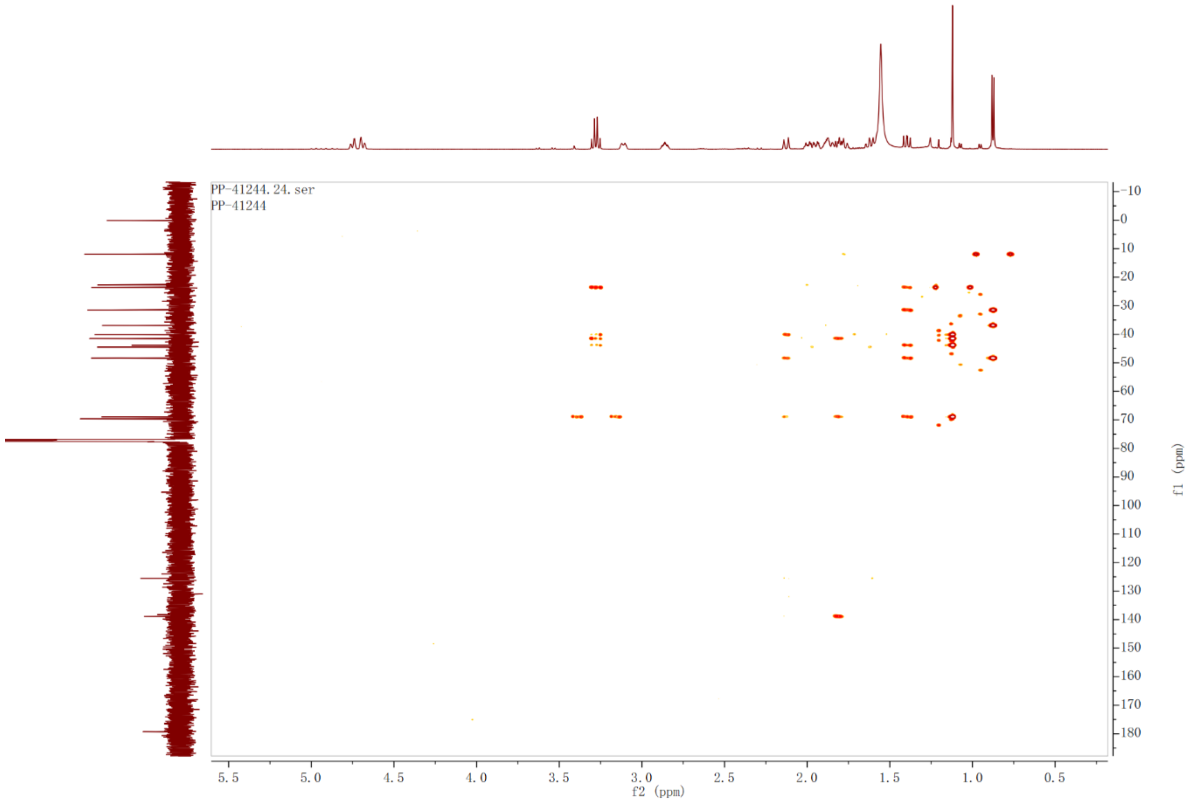


**Figure S12.** HMBC ($CDCl_3$) spectrum of compound **2**

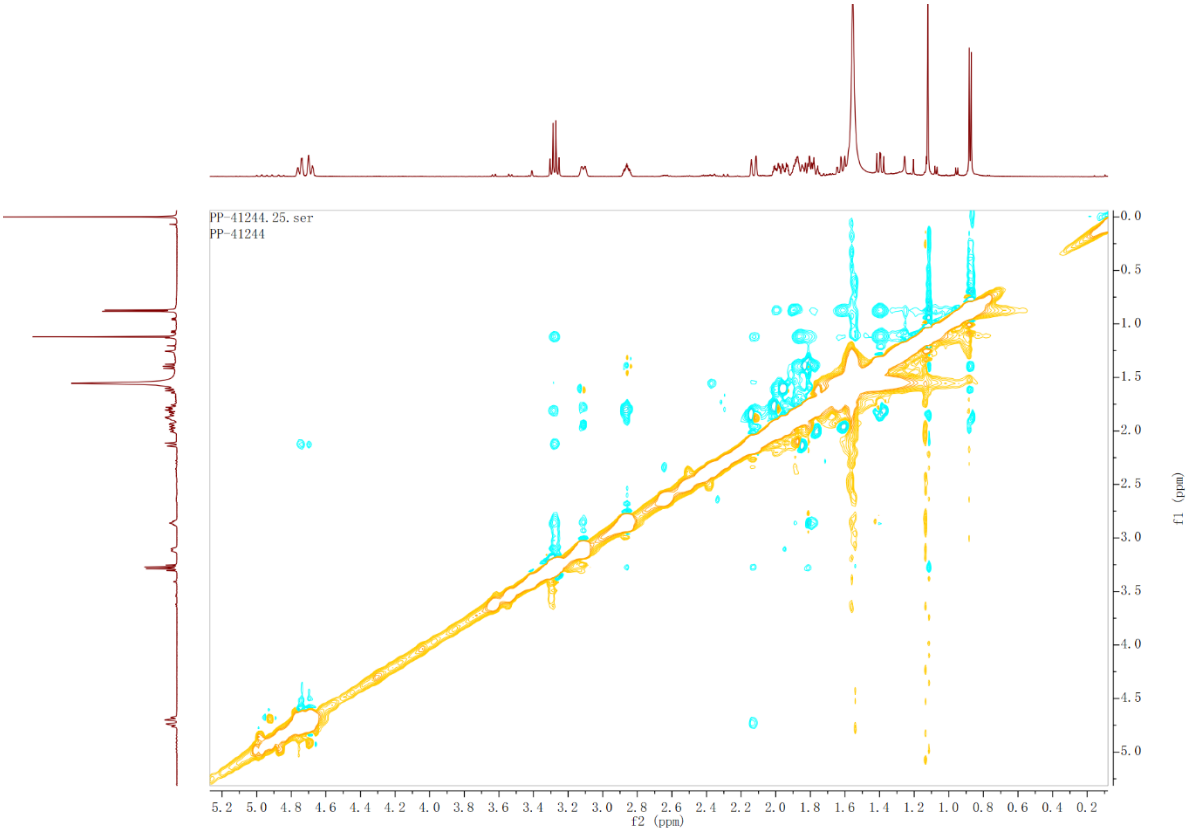


**Figure S13.** NOESY ($CDCl_3$) spectrum of compound **2**

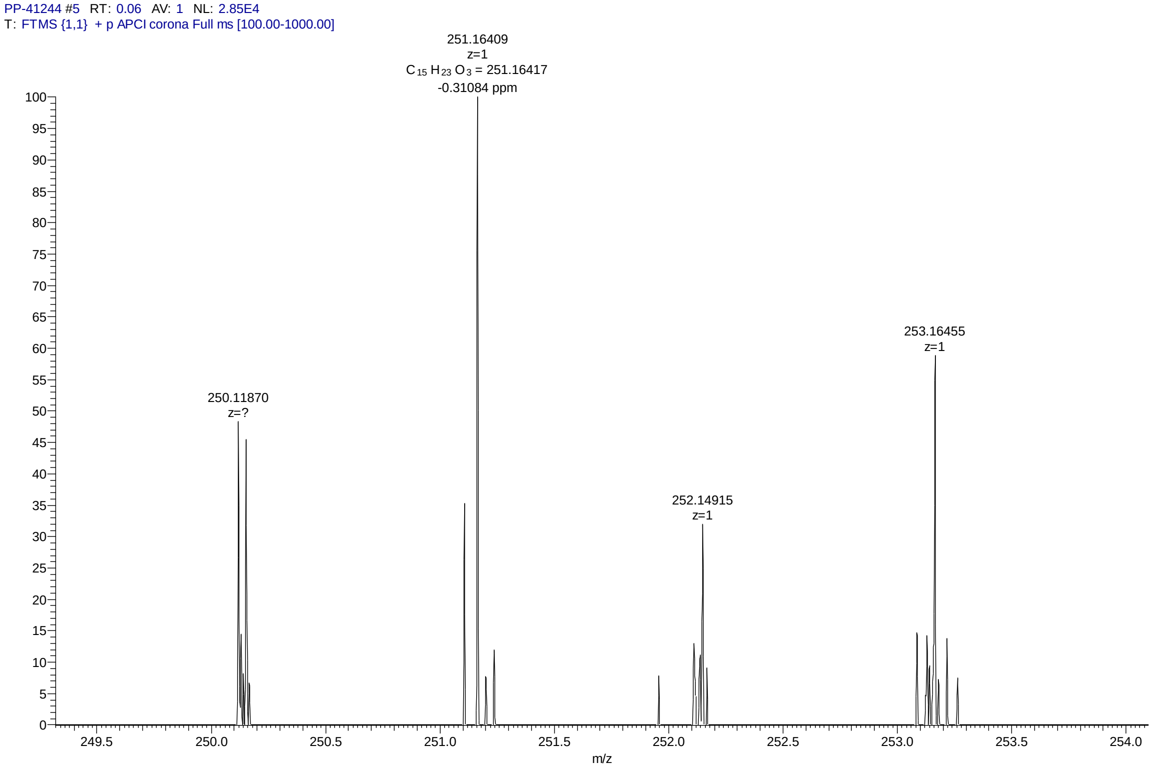


**Figure S14.** HR-APCI-MS spectrum of compound **2**

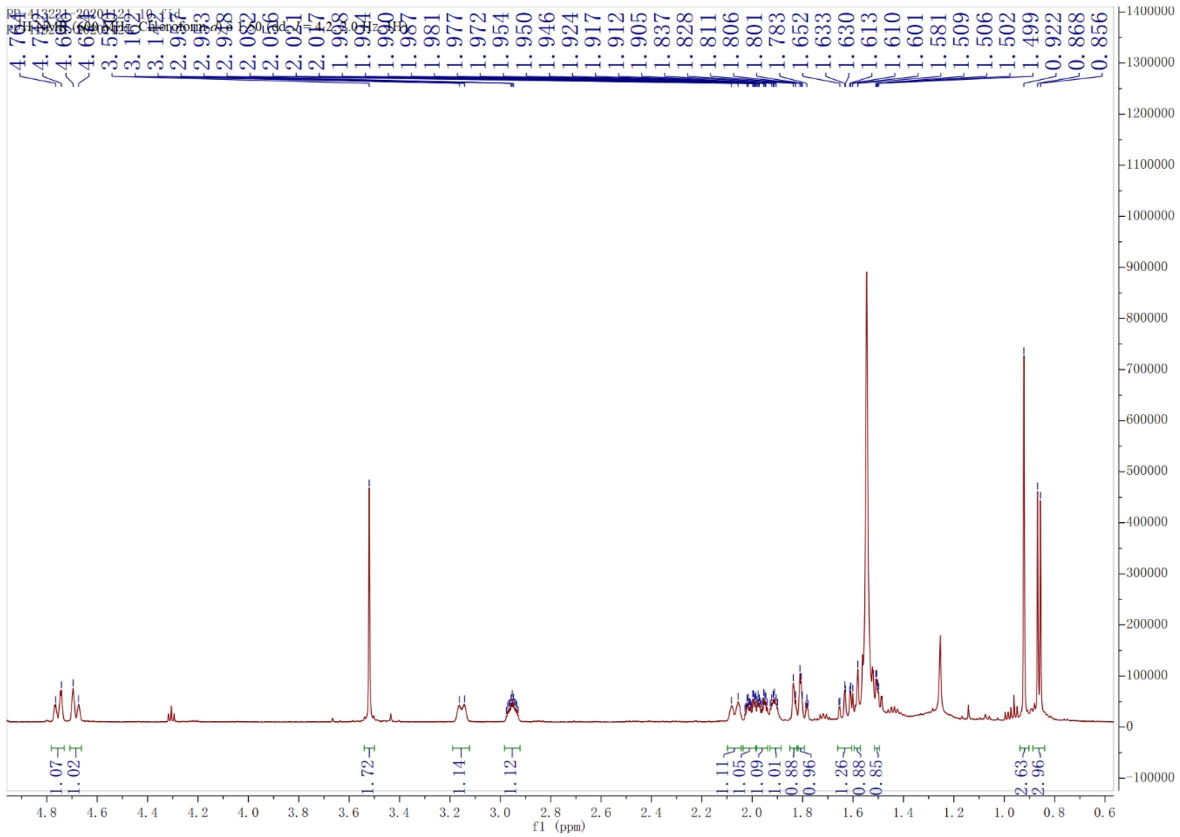

**Figure S15.** $^{1}$H NMR (600 MHz, $CDCl_3$) spectrum of compound **3**

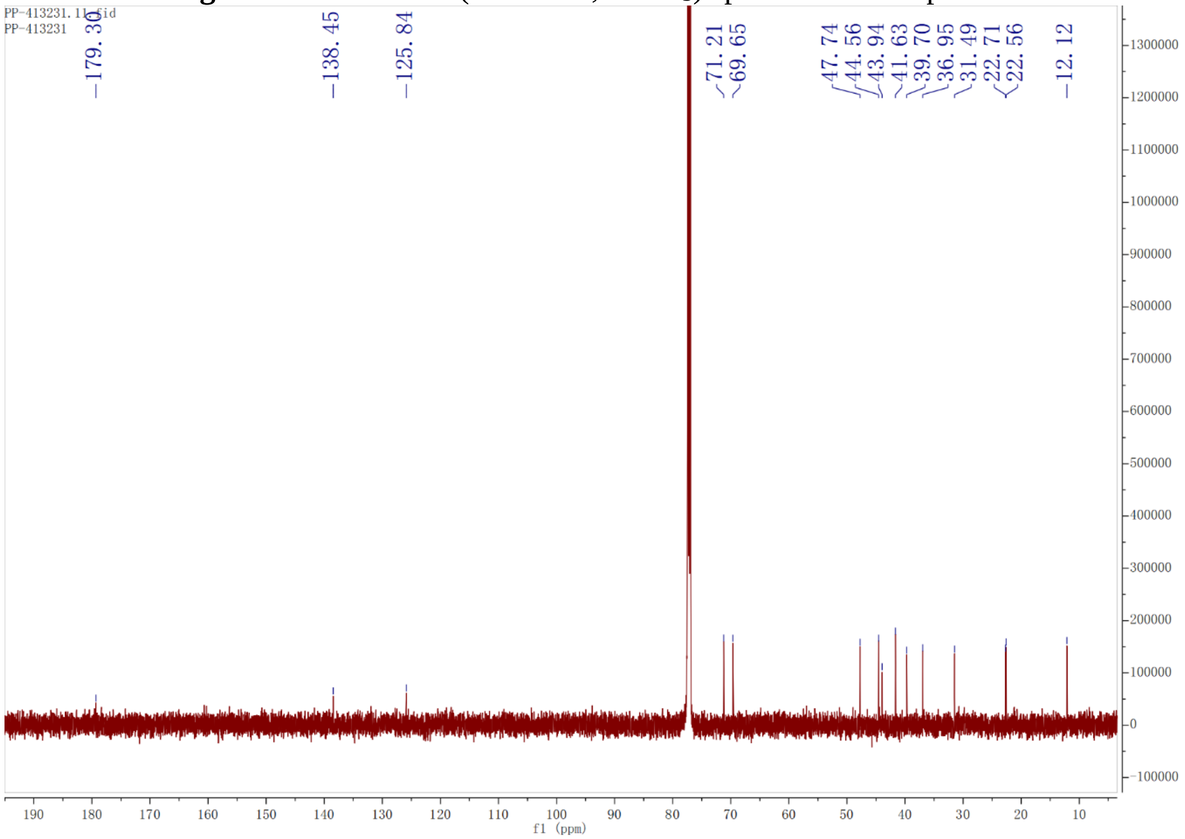

**Figure S16.** $^{13}$C NMR (150 MHz, $CDCl_3$) spectrum of compound **3**

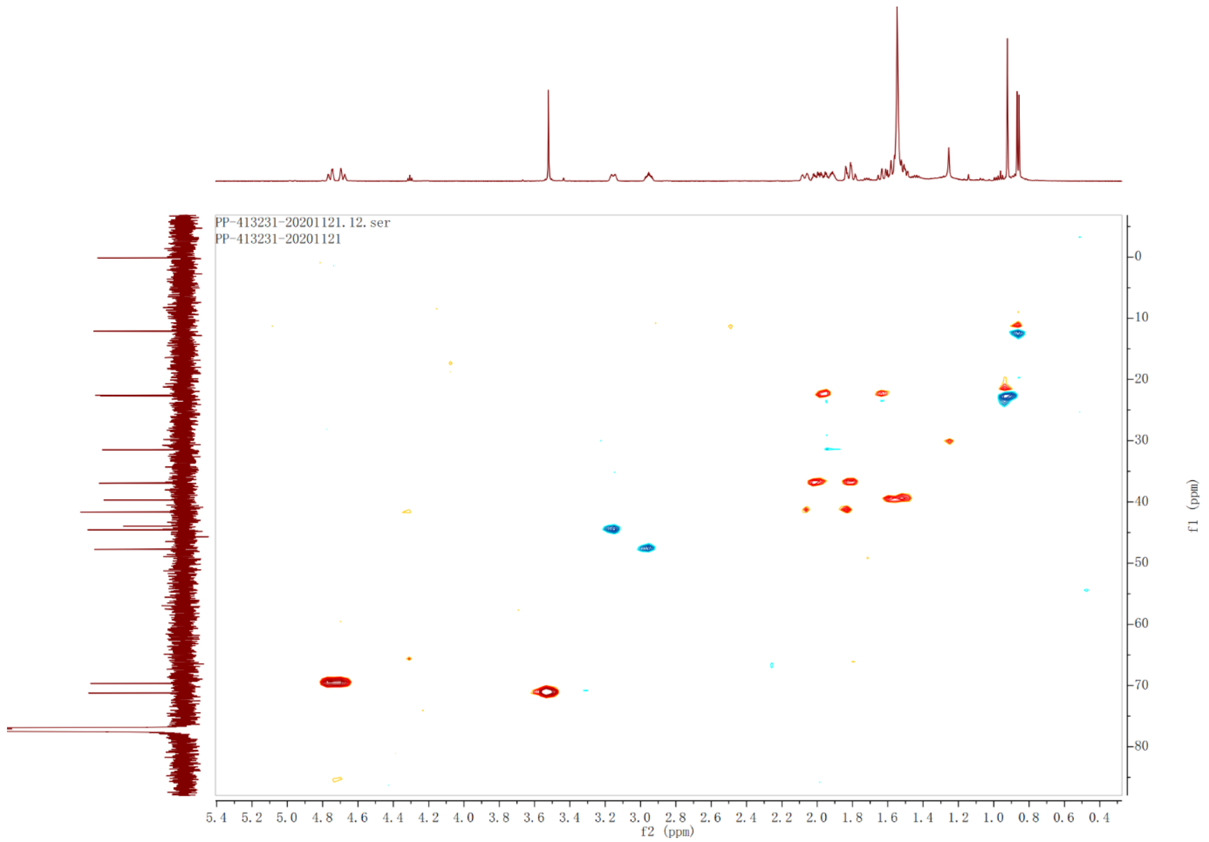


**Figure S17.** HSQC ($CDCl_3$) spectrum of compound **3**

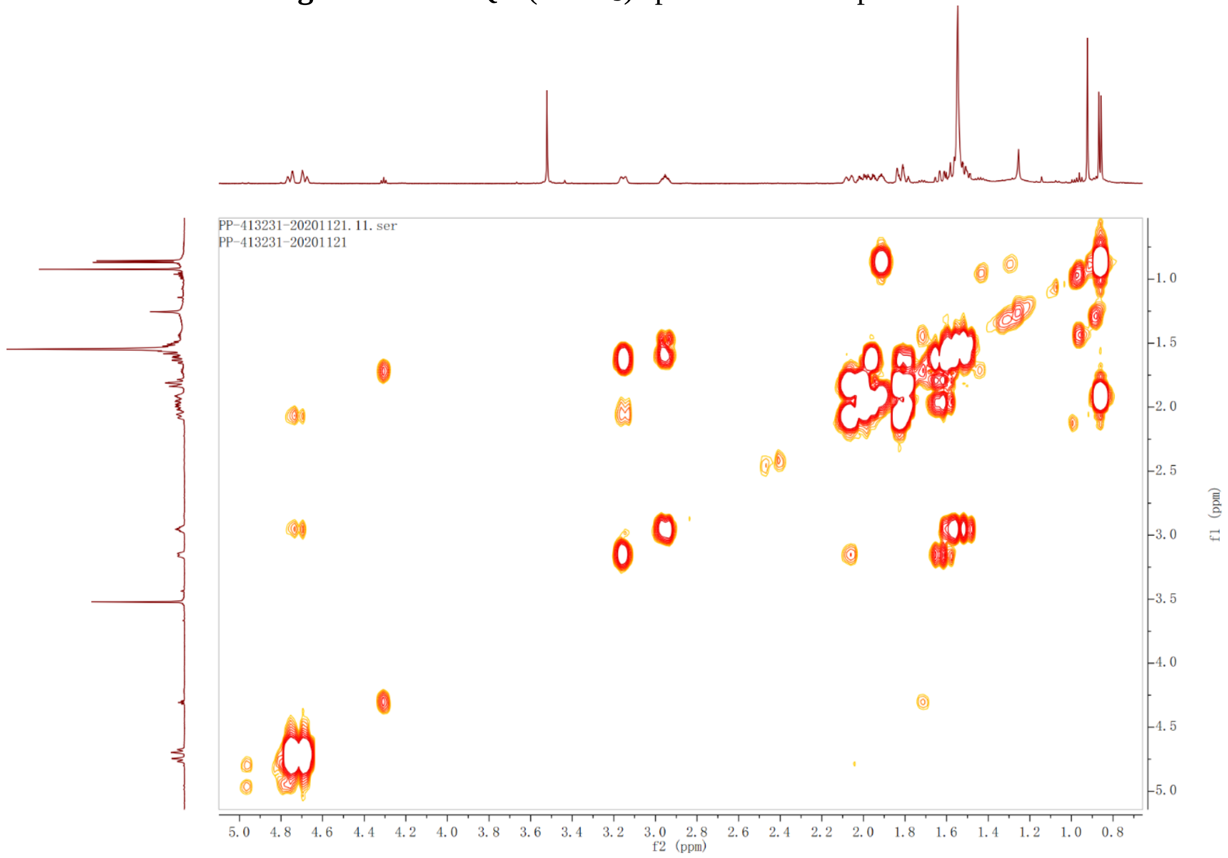

**Figure S18.** COSY ($CDCl_3$) spectrum of compound **3**

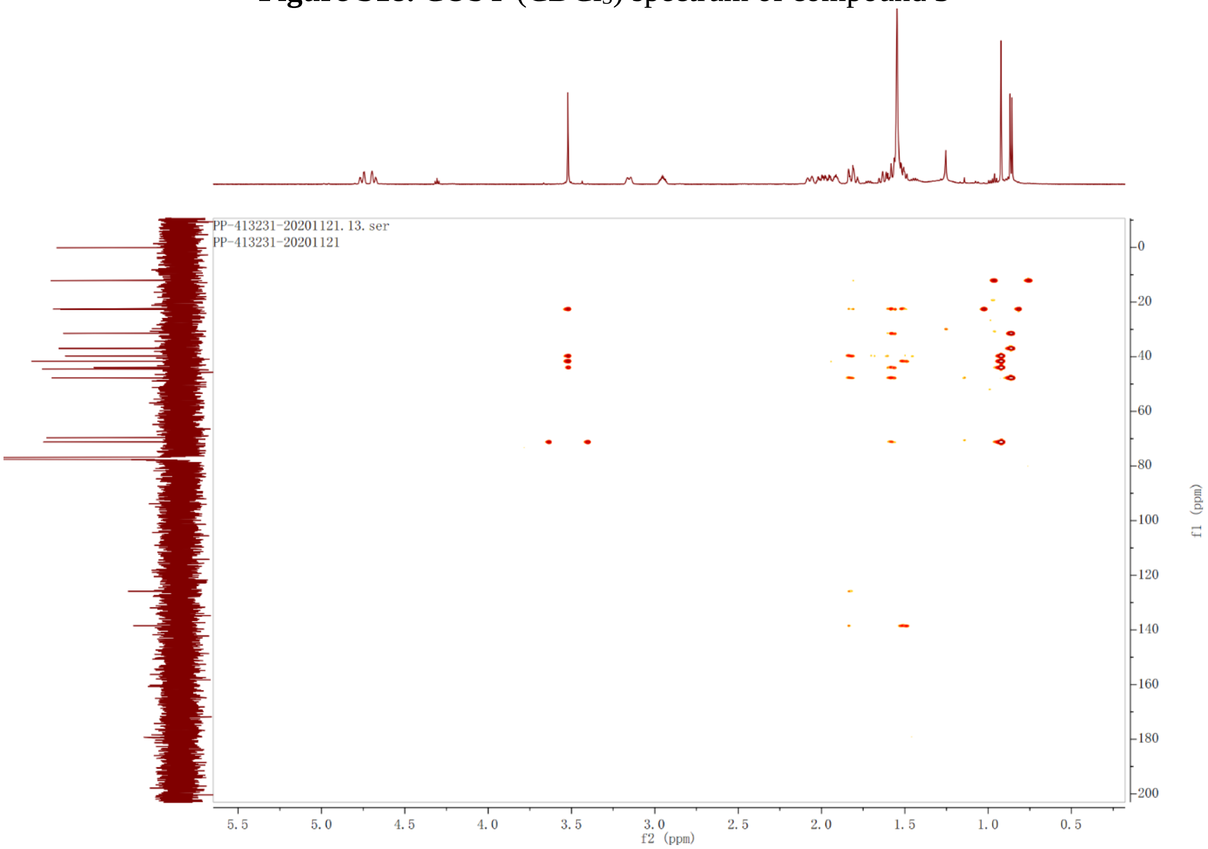


**Figure S19.** HMBC ($CDCl_3$) spectrum of compound **3**

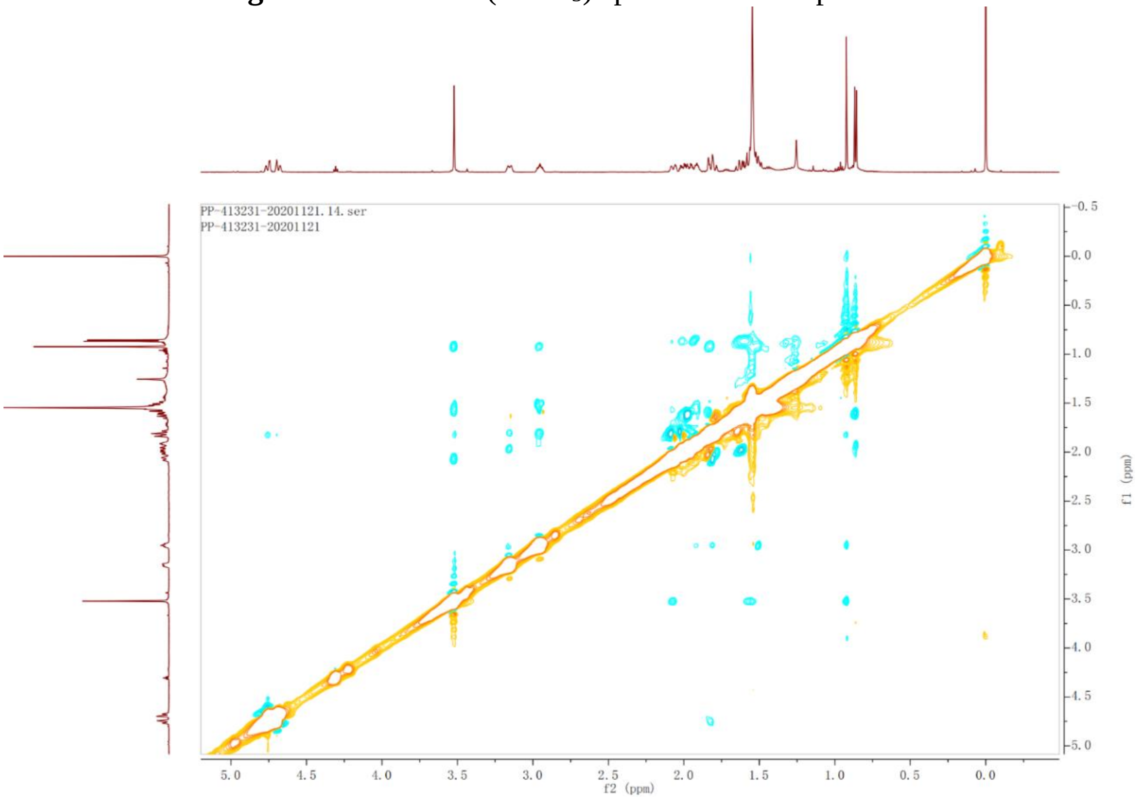


**Figure S20.** NOESY ($CDCl_3$) spectrum of compound **3**

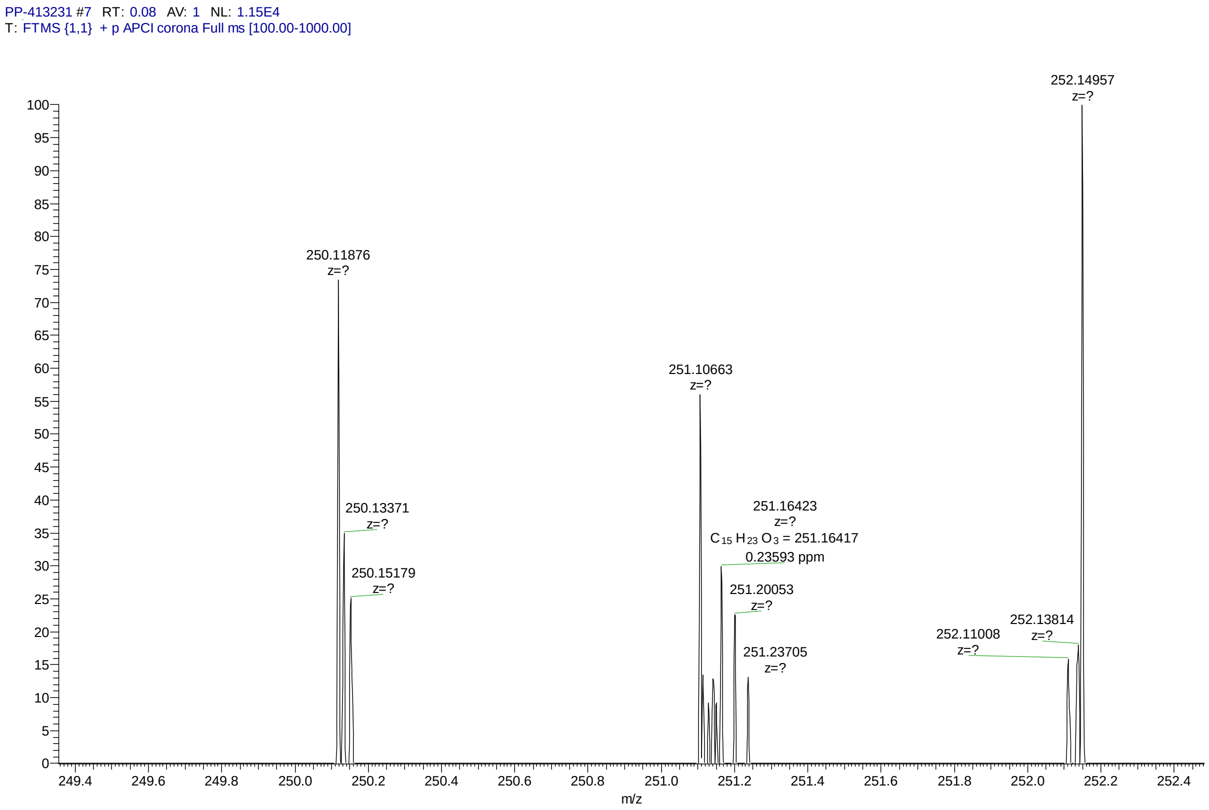


**Figure S21.** HR-APCI-MS spectrum of compound **3**

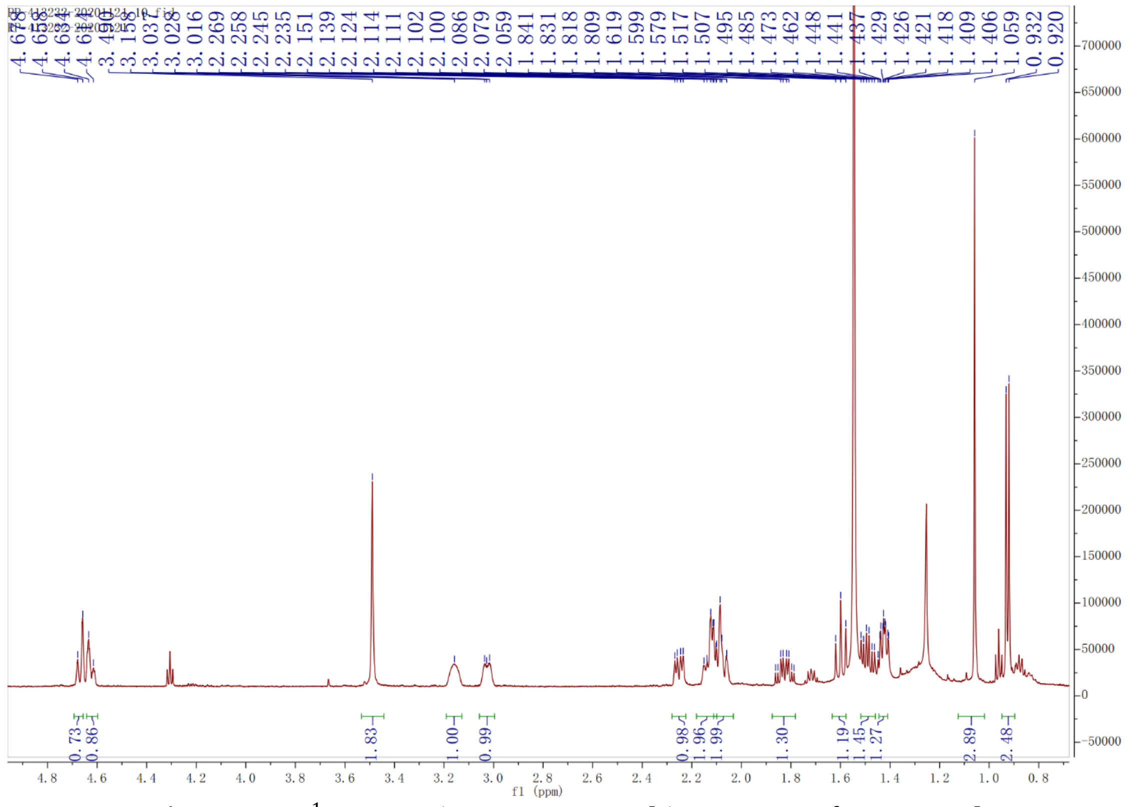


**Figure S22.** $^{1}H$ NMR (600 MHz, $CDCl_3$) spectrum of compound **4**

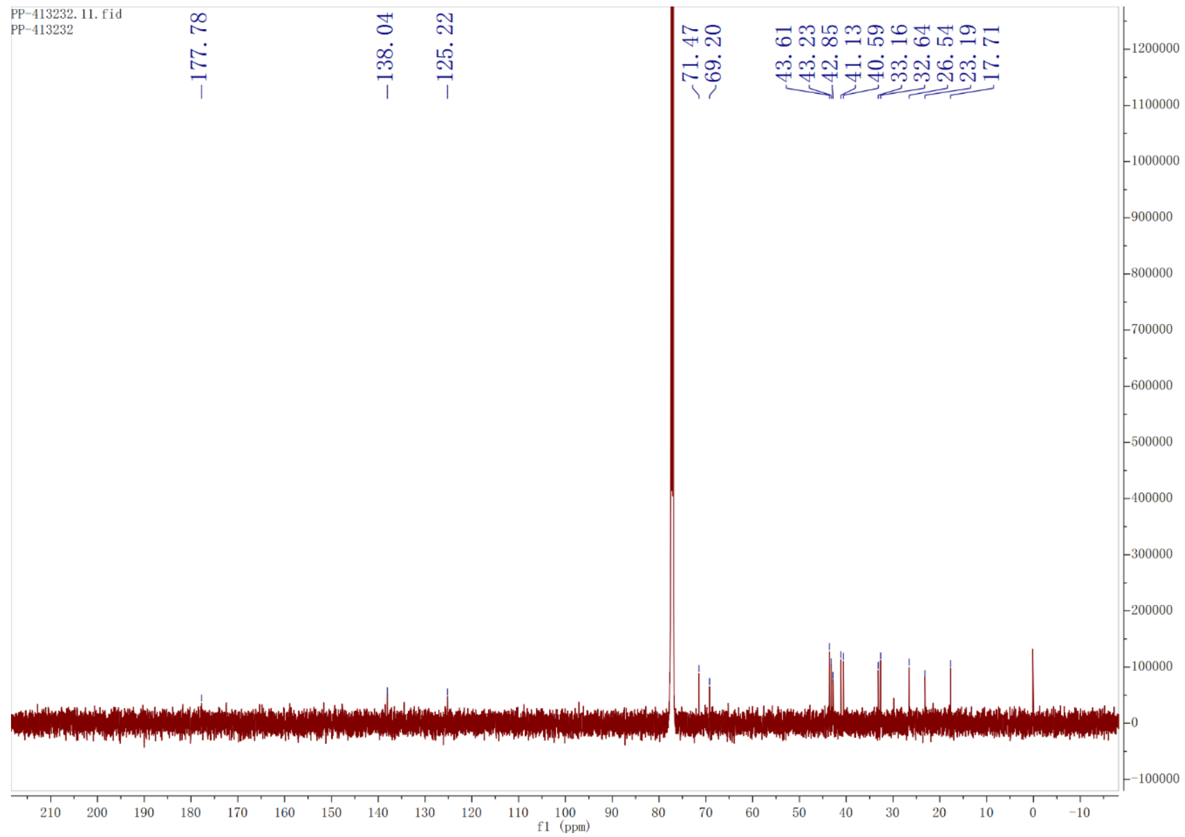


**Figure S23.** $^{13}$C NMR (150 MHz, $CDCl_3$) spectrum of compound **4**

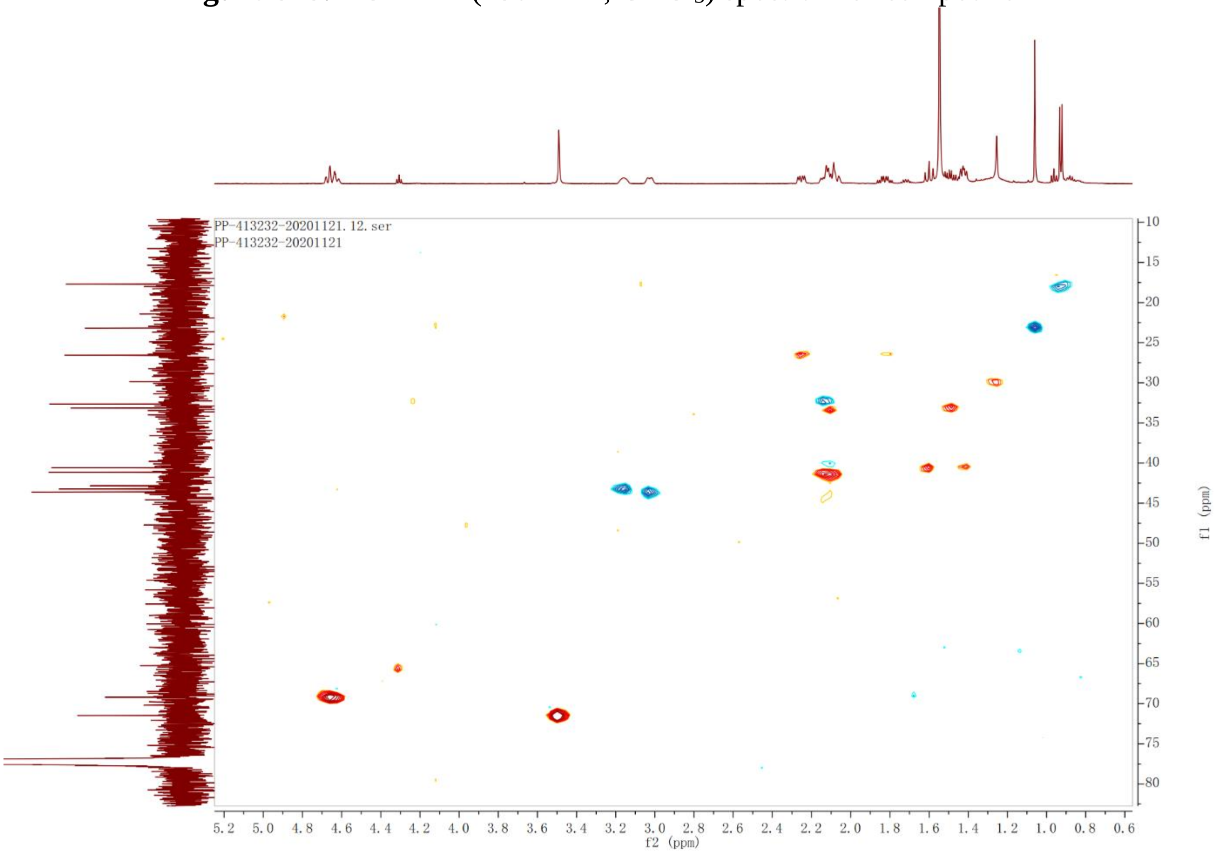


**Figure S24.** HSQC ($CDCl_3$) spectrum of compound **4**

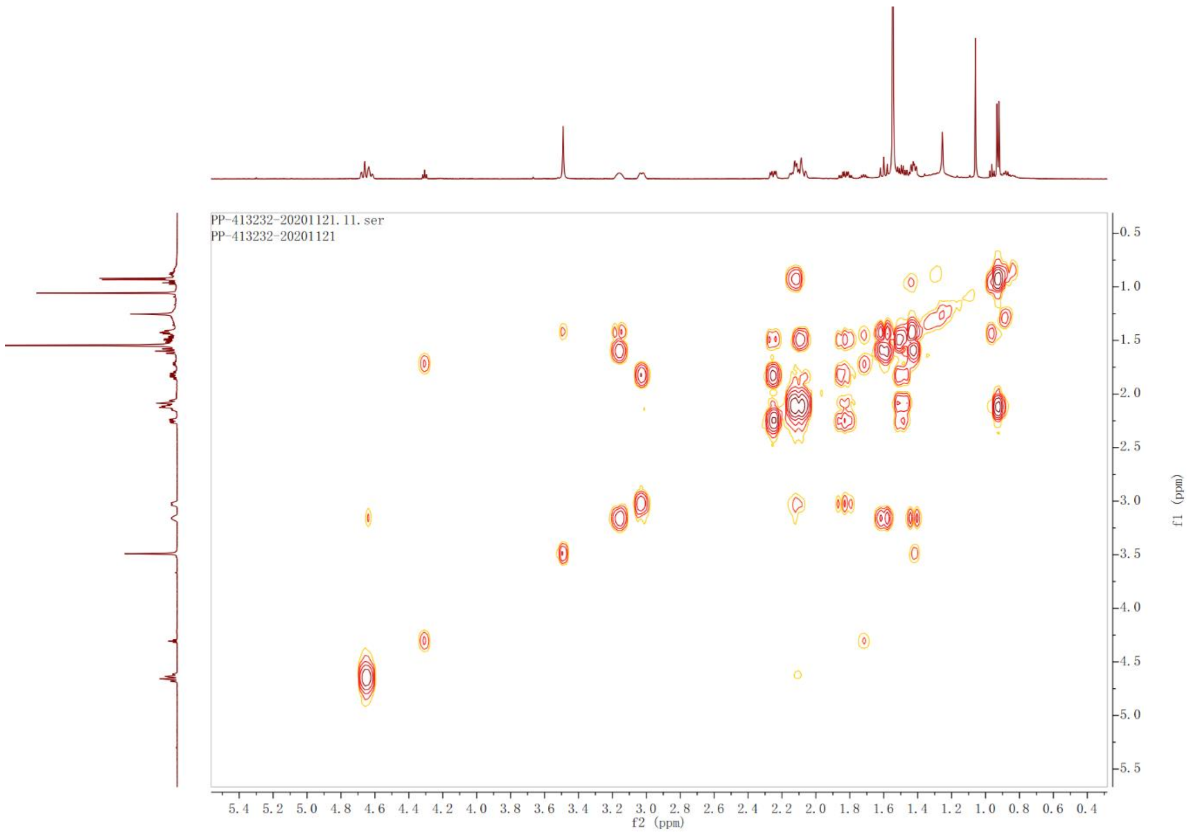


**Figure S25.** COSY ($CDCl_3$) spectrum of compound **4**

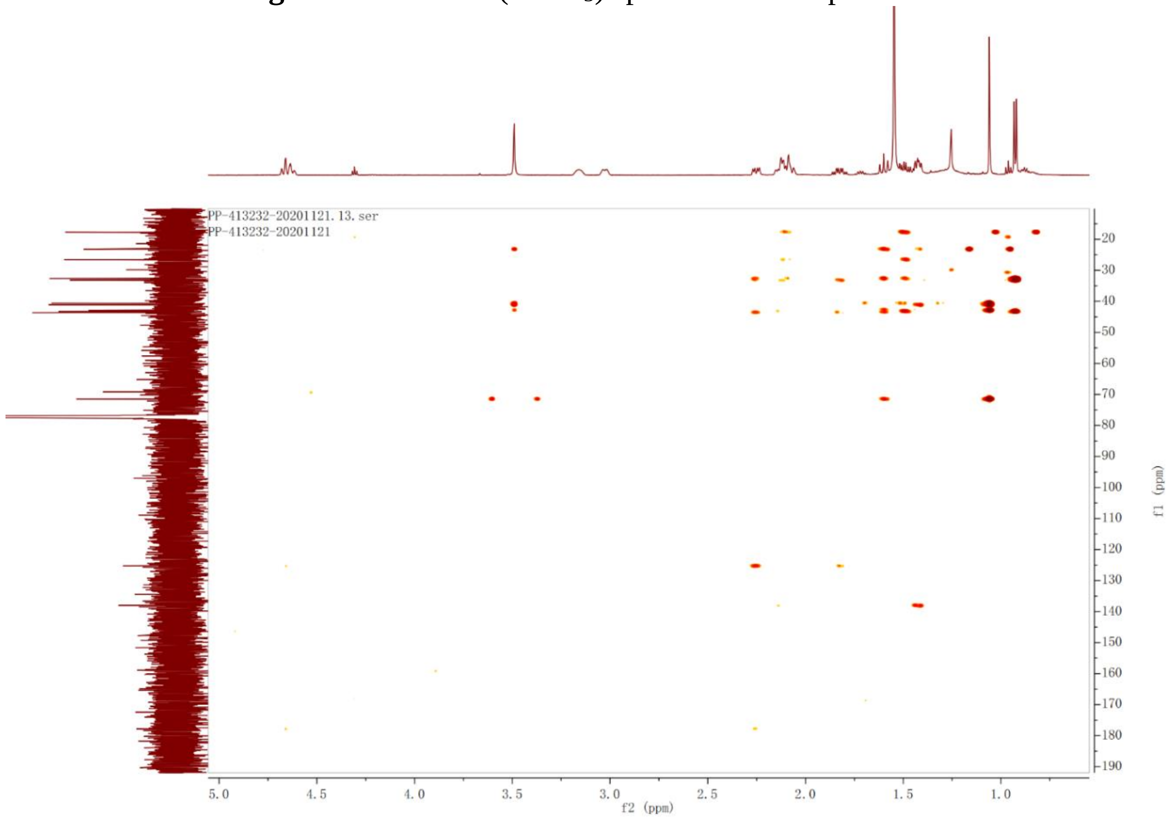


**Figure S26.** HMBC ($CDCl_3$) spectrum of compound **4**

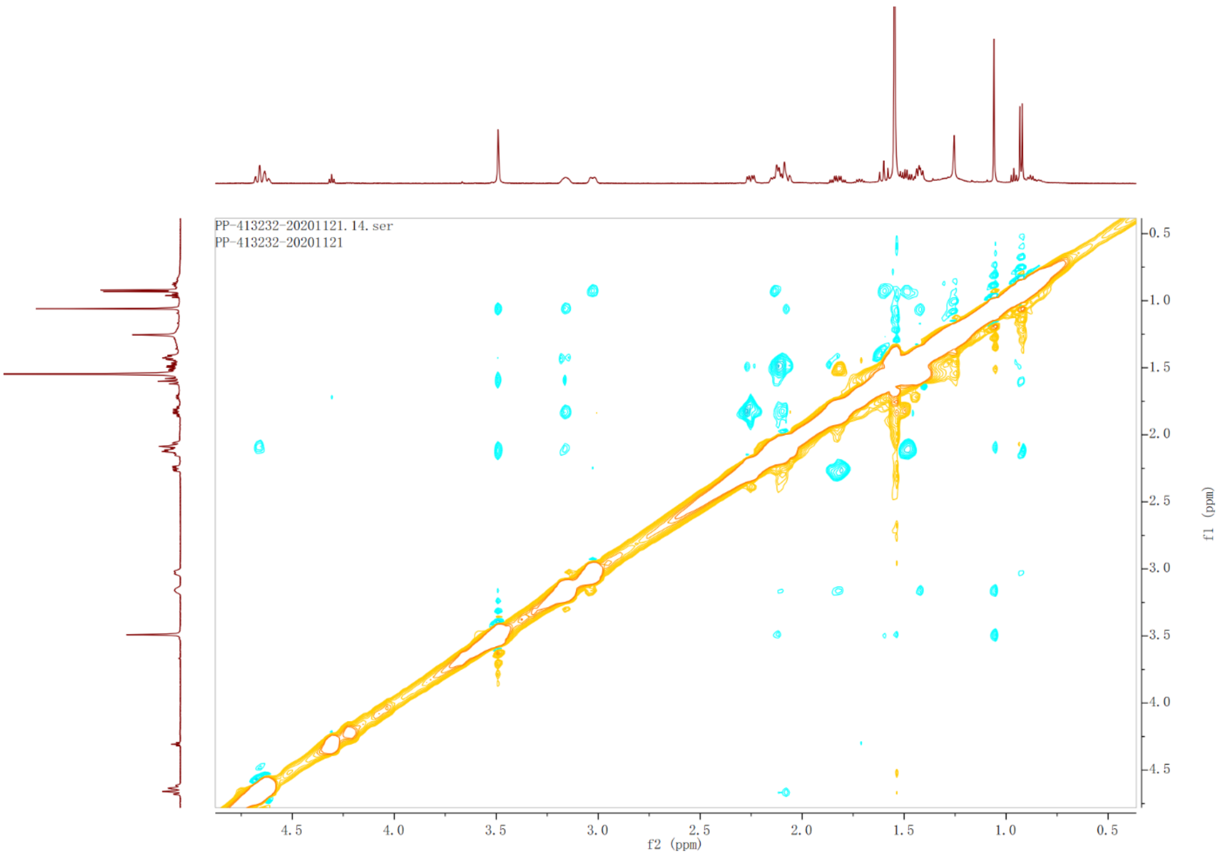


**Figure S27.** NOESY ($CDCl_3$) spectrum of compound **4**

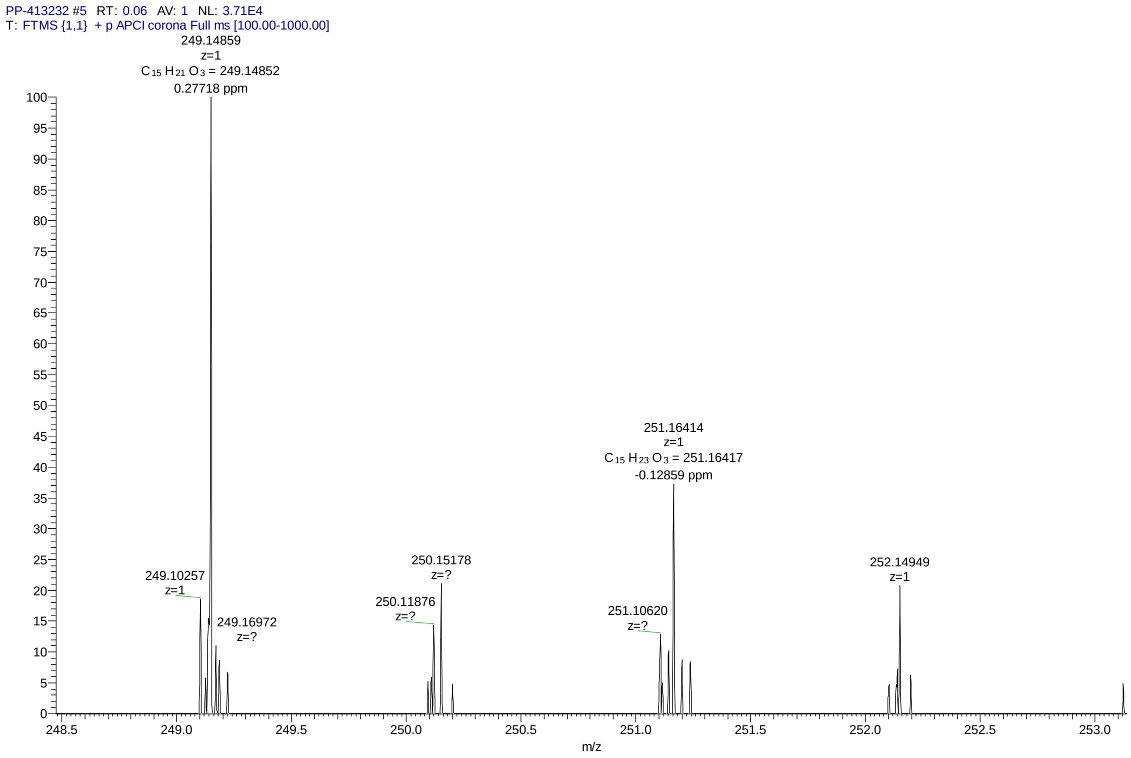


**Figure S28.** HR-APCI-MS spectrum of compound **4**

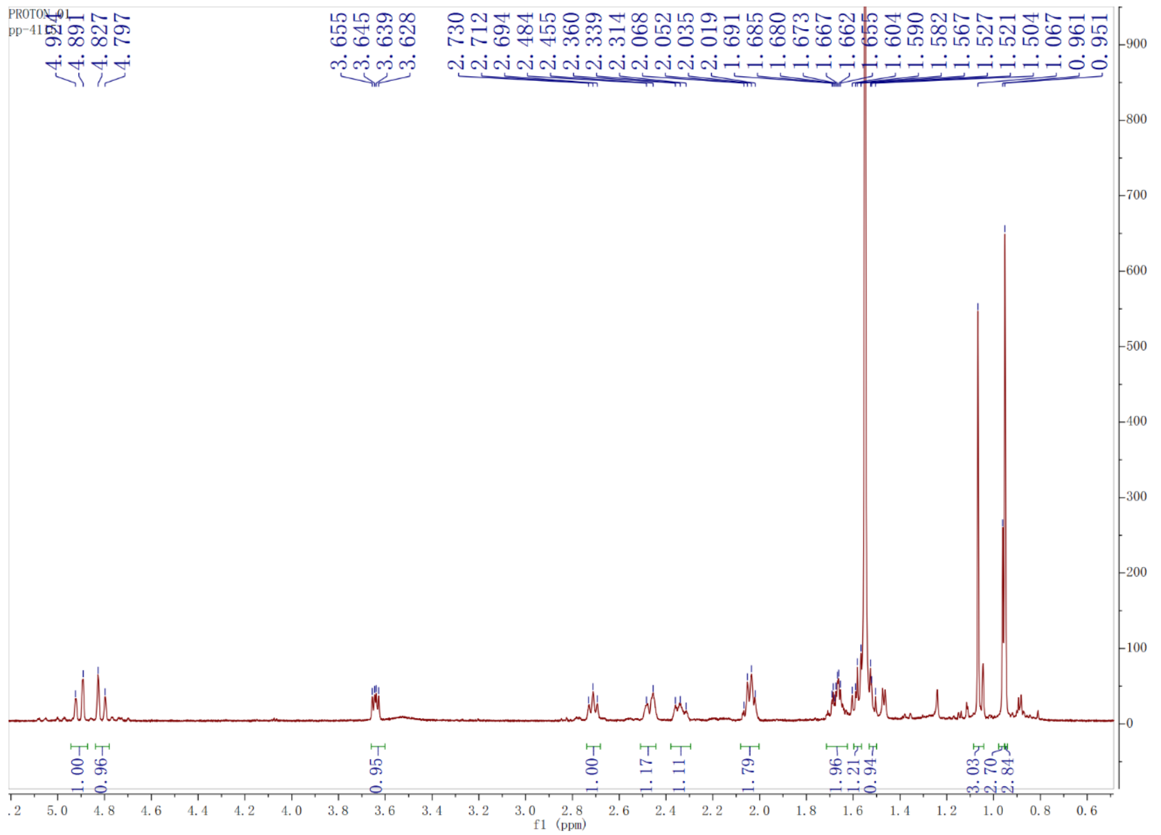

**Figure S29.** $^{1}H$ NMR (600 MHz, $CDCl_3$) spectrum of compound **5**

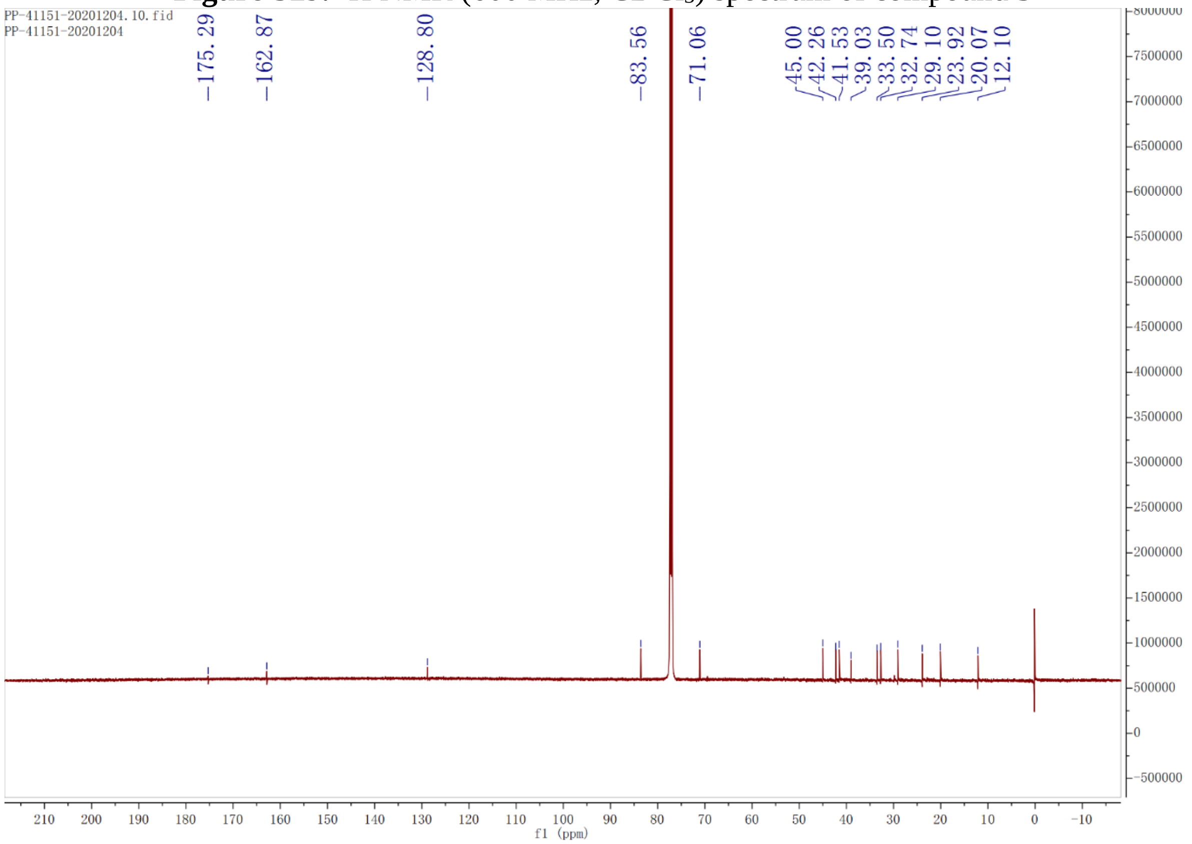

**Figure S30.** $^{13}C$ NMR (150 MHz, $CDCl_3$) spectrum of compound **5**

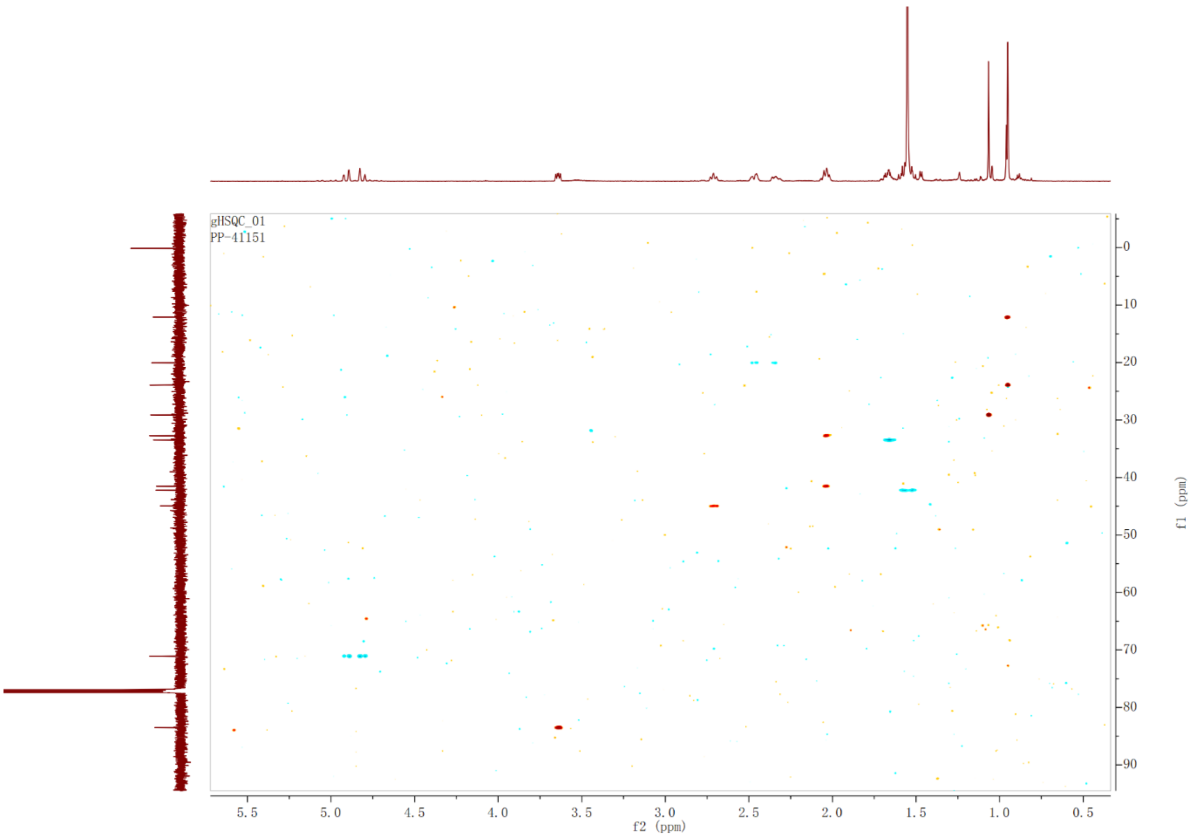


**Figure S31.** HSQC ($CDCl_3$) spectrum of compound **5**

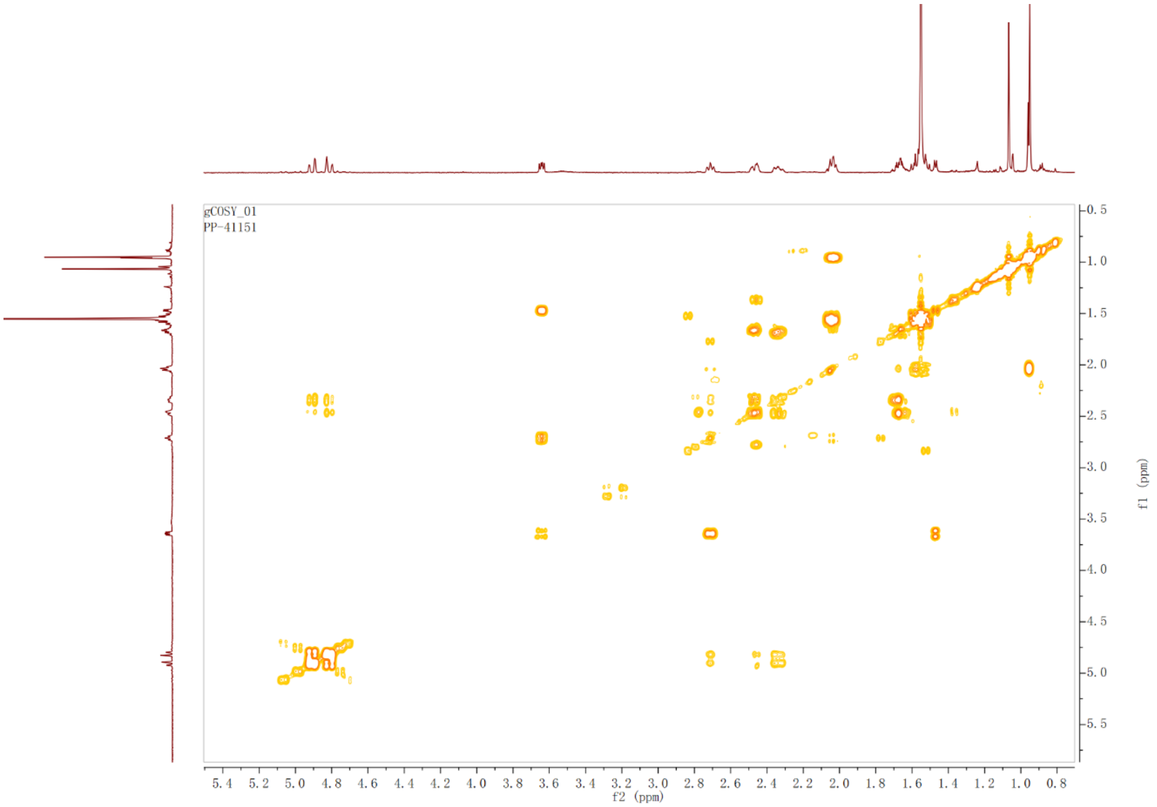


**Figure S32.** COSY ($CDCl_3$) spectrum of compound **5**

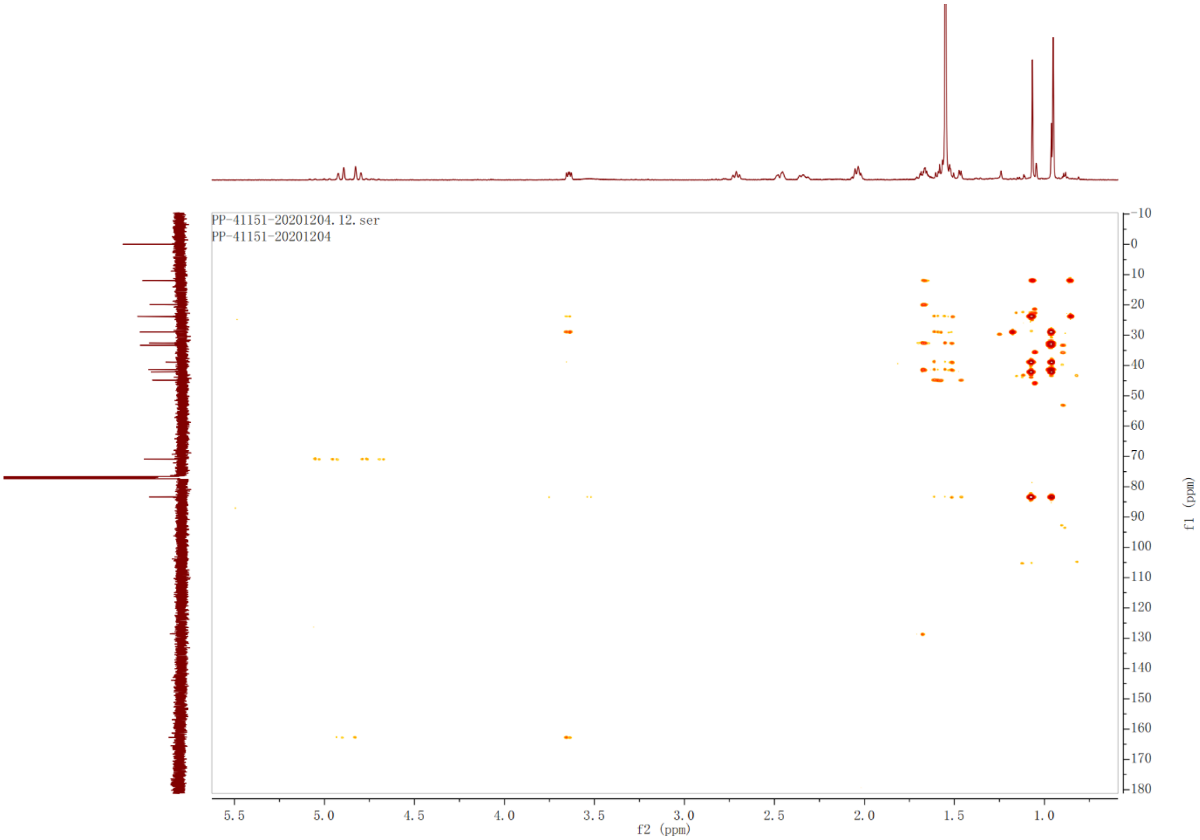


**Figure S33.** HMBC ($CDCl_3$) spectrum of compound **5**

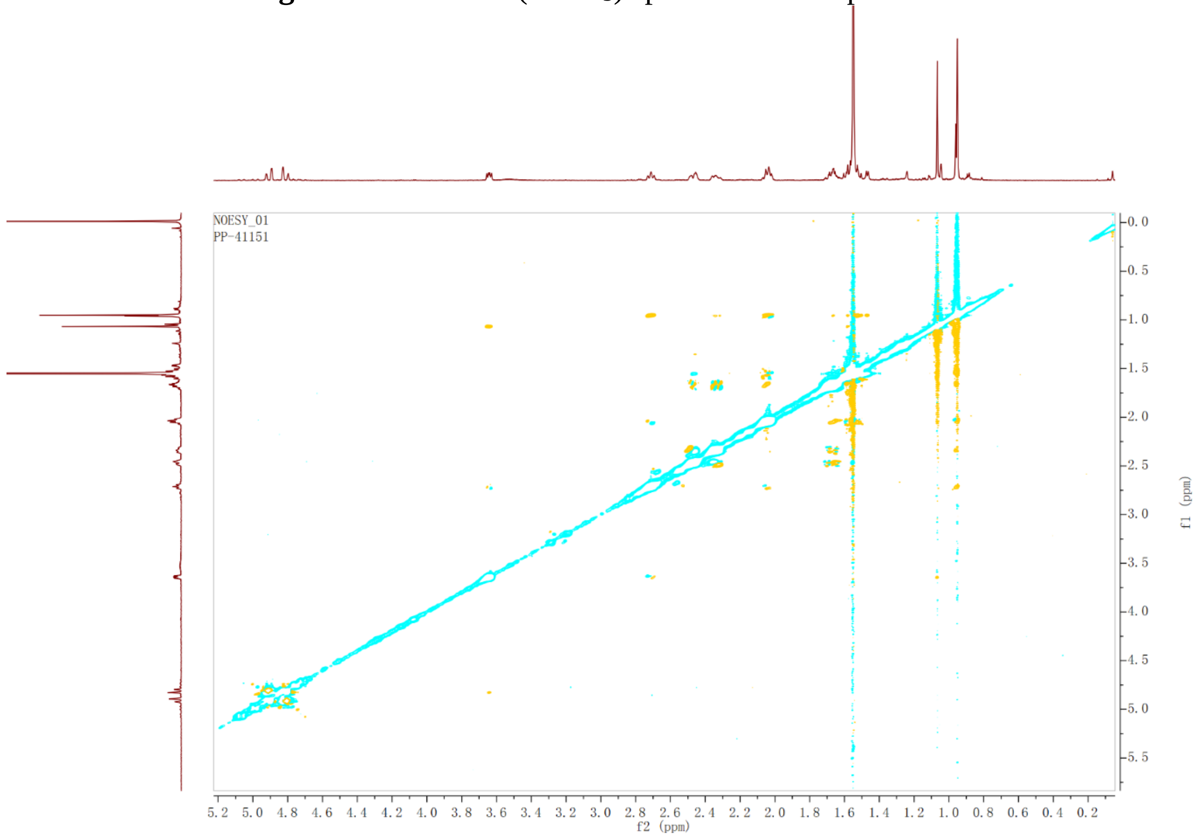


**Figure S34.** NOESY ($CDCl_3$) spectrum of compound **5**

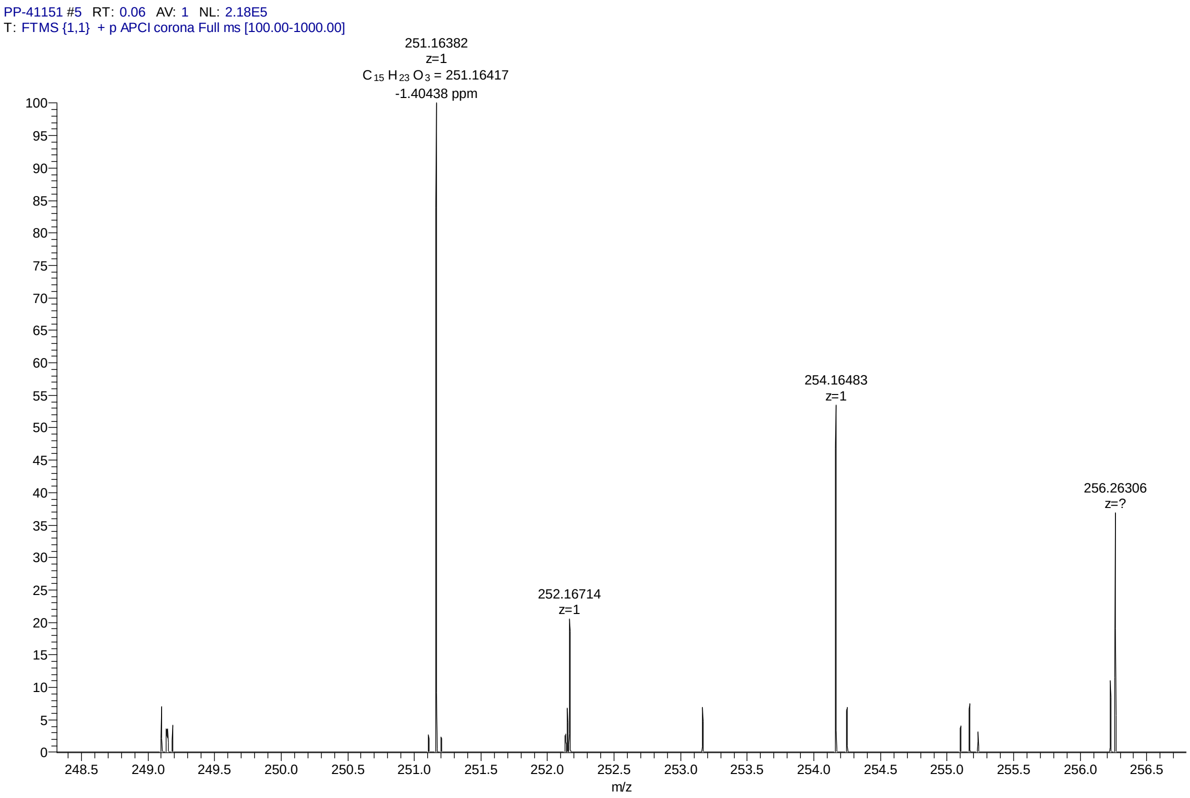


**Figure S35.** HR-APCI-MS spectrum of compound **5**

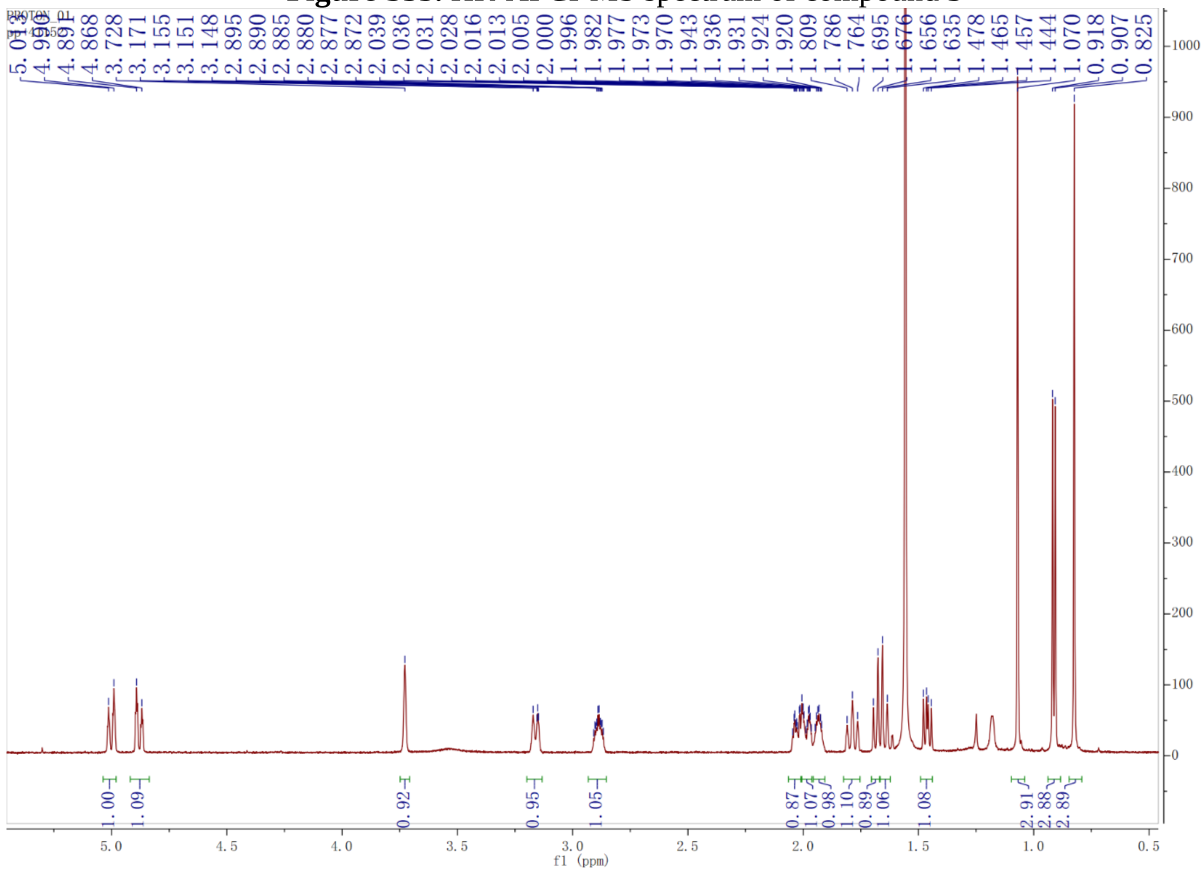


**Figure S36.** $^{1}$H NMR (600 MHz, $CDCl_3$) spectrum of compound **6**

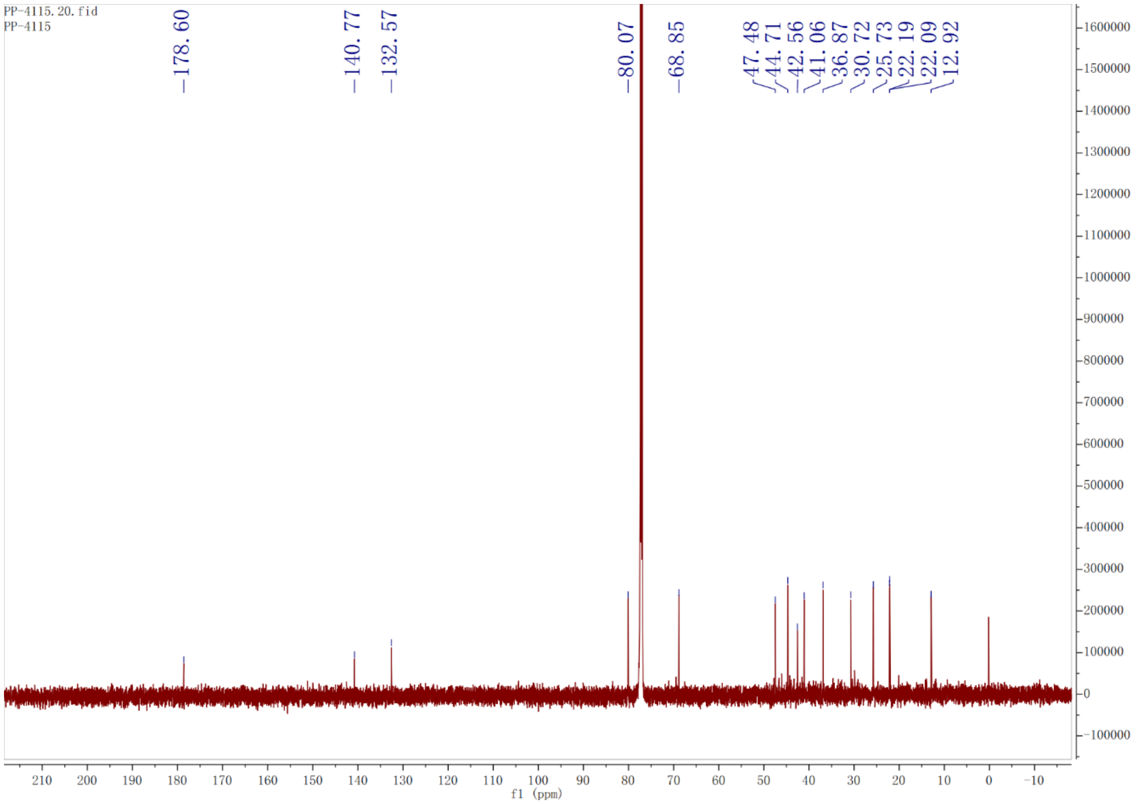


**Figure S37.** $^{13}$C NMR (150 MHz, $CDCl_3$) spectrum of compound **6**

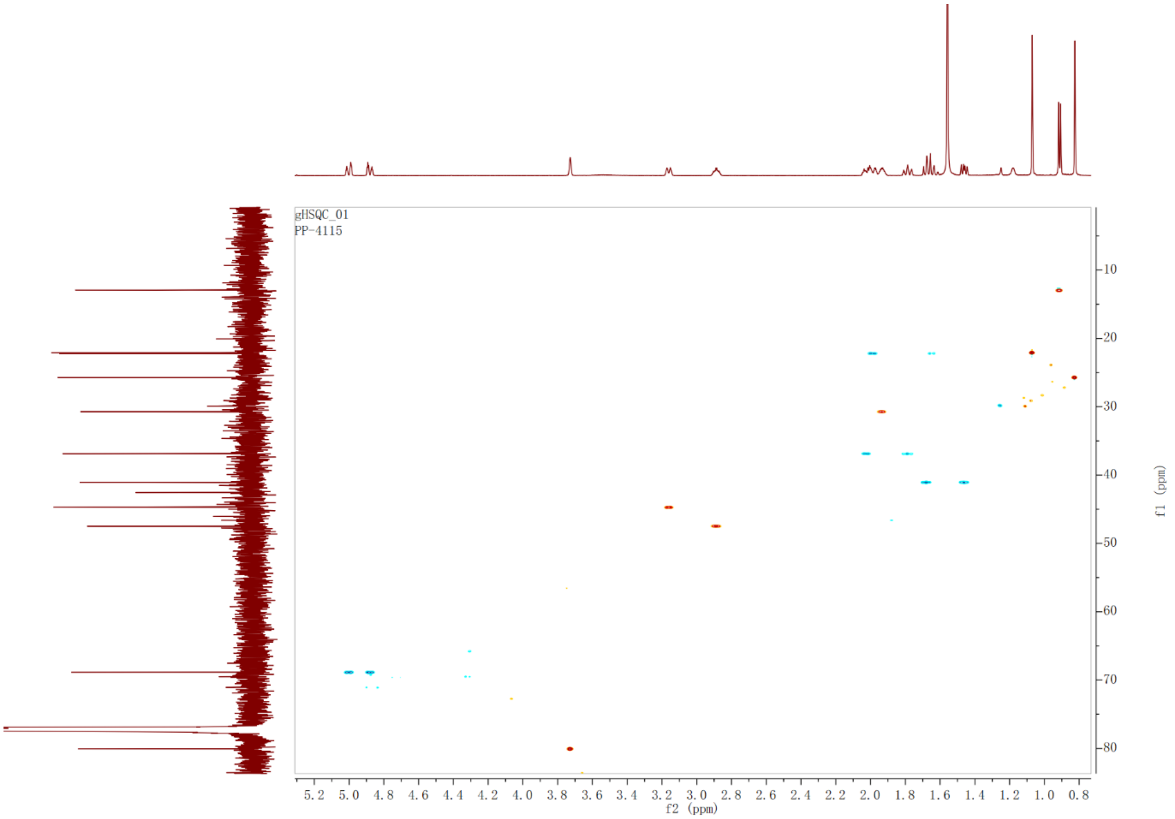


**Figure S38.** HSQC ($CDCl_3$) spectrum of compound **6**

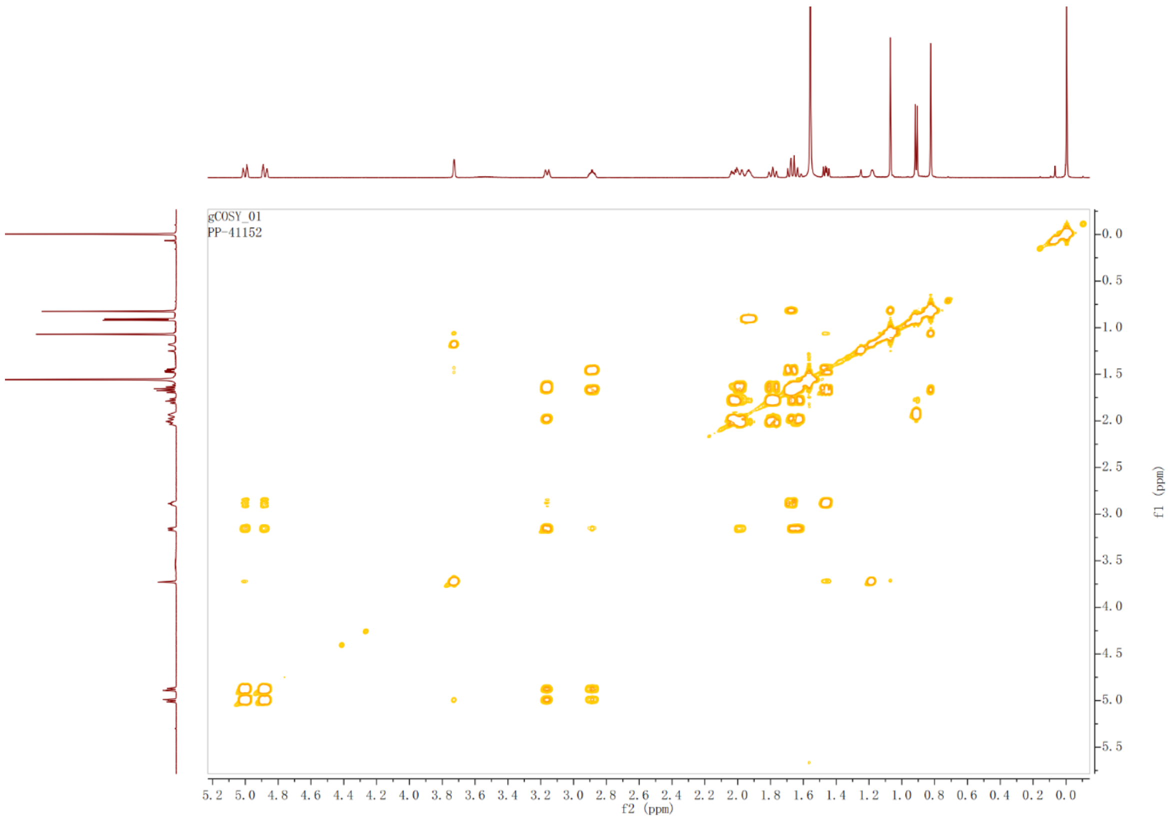


**Figure S39.** COSY ($CDCl_3$) spectrum of compound **6**

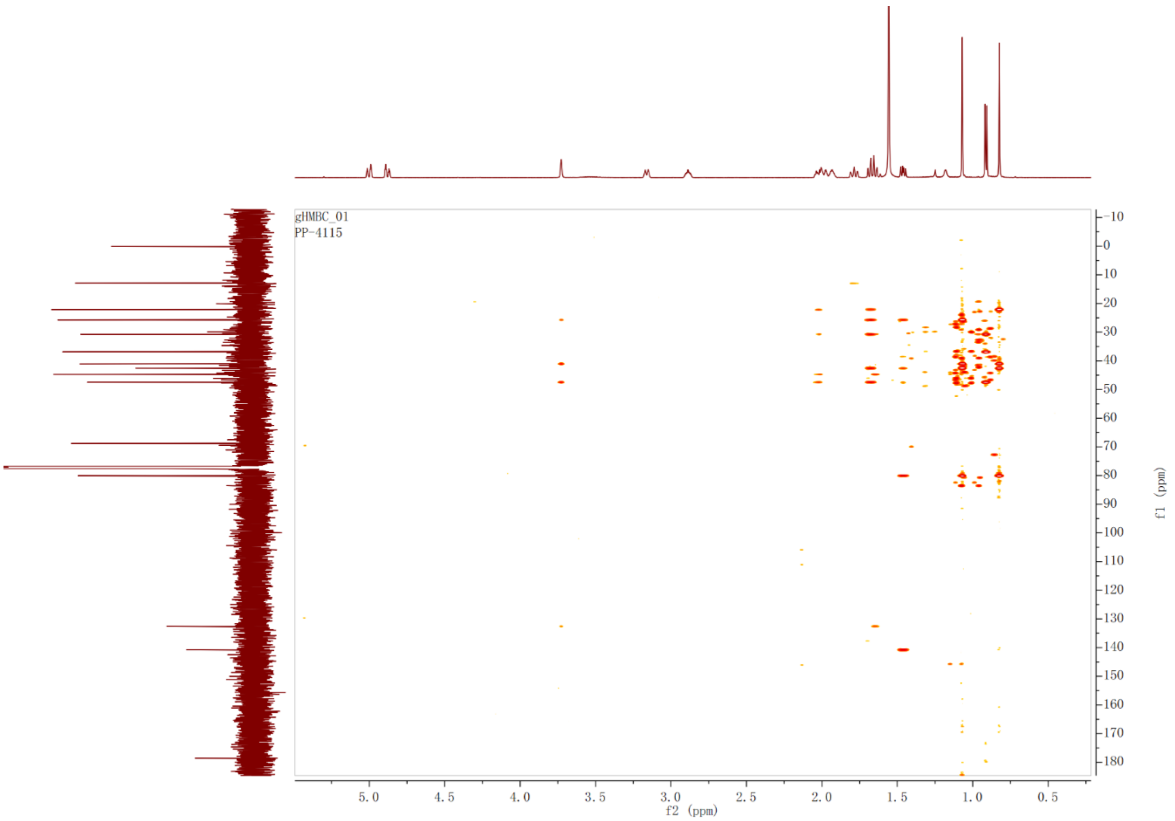


**Figure S40.** HMBC ($CDCl_3$) spectrum of compound **6**

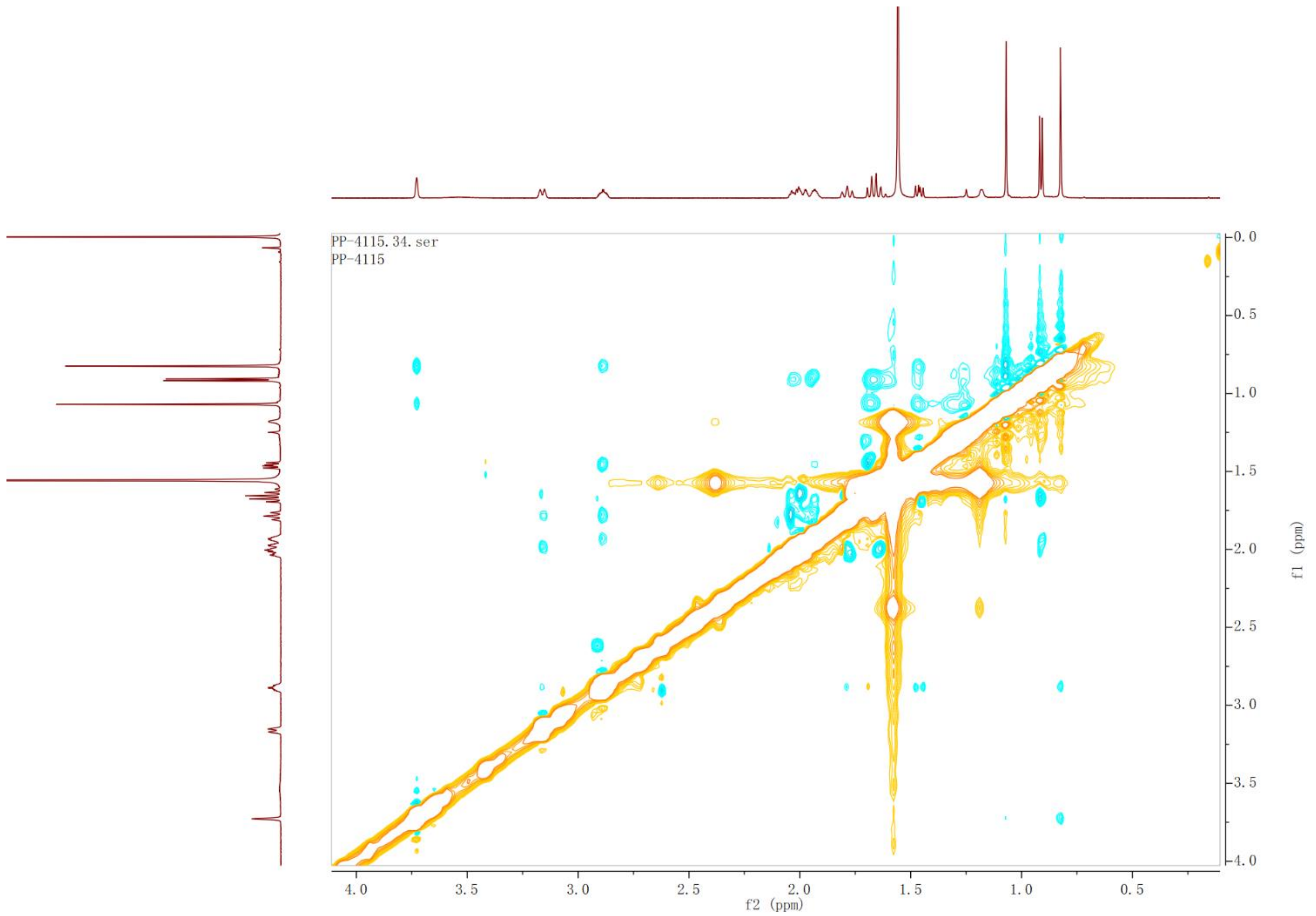


**Figure S41.** NOESY ($CDCl_3$) spectrum of compound **6**

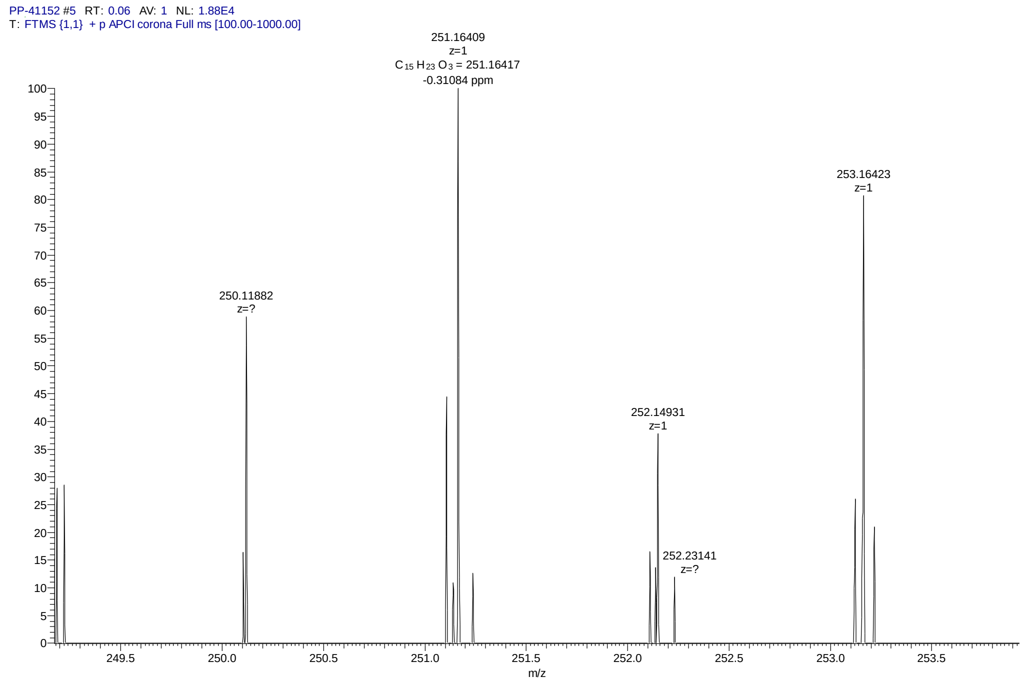


**Figure S42.** HR-APCI-MS spectrum of compound **6**

**Table S1.** Inhibition rates of tested cell lines of compounds **8** and **9**.

| Compounds | Concentration (μM) | MDA-MB-231 | A549 | HCT116 | HepG2 | PANC-1 |
|---|---|---|---|---|---|---|
| **8** | 80 | 79±1.5 | 77±2.3 | 87±1.8 | 88±2.7 | 61±3.1 |
| | 40 | 74±0.78 | 44±1.8 | 85±1.8 | 86±1.6 | 46±2.5 |
| | 20 | 28±0.82 | 7.0±1.5 | 39±1.3 | 48±4.3 | 21±2.2 |
| | 10 | 14±1.7 | 3.1±1.5 | 9.9±2.3 | 8.8±1.1 | 6.5±1.2 |
| | 5 | -0.13±0.40 | 0.12±0.87 | -3.2±1.5 | -4.1±0.22 | -3.2±1.6 |
| **9** | 80 | 85±1.3 | 79±2.4 | 89±1.6 | 86±4.1 | 70±2.1 |
| | 40 | 79±1.5 | 52±7.7 | 88±1.1 | 88±2.5 | 57±1.8 |
| | 20 | 31±0.83 | 26±3.0 | 43±0.57 | 43±1.6 | 25±1.6 |
| | 10 | 19±1.1 | 9.0±1.8 | 18±2.9 | 20±5.3 | 8.5±2.8 |
| | 5 | 20±3.2 | 12±1.3 | 8.7±3.8 | 13±2.0 | -2.3±3.1 |

**Table S2.** Inhibition rates of tested marine fouling bacteria of compounds **1−12**.

| Comps (20 μM) | *P. fulva* | *A. hydrophila* | *A. salmonicida* | *V. anguillarum* | *V. harveyi* | *P. halotolerans* | *P. angustum* | *E. cloacae* | *E. hormaechei* |
|---|---|---|---|---|---|---|---|---|---|
| **1** | 5.1±2.15 | -6.4±0.25 | 0.70±0.20 | -5.7±3.0 | -1.3±0.40 | -1.3±2.2 | -9.5±0.90 | -9.9±2.8 | -1.7±3.1 |
| **2** | 6.4±0.25 | -6.7±0.65 | -4.2±1.1 | -5.7±2.3 | -12.4±2.0 | -1.1±2.9 | -9.8±1.4 | -1.1±1.4 | -4.8±4.1 |
| **3** | 2.5±0.10 | -3.7±0.40 | -3.7±0.80 | -5.0±0.75 | -9.3±1.1 | -8.9±2.1 | -5.6±1.4 | -6.8±1.1 | -2.6±2.1 |
| **4** | 5.1±0.25 | 0.41±0.60 | -4.8±0.25 | -2.5±1.2 | -8.6±1.3 | -4.0±1.4 | -0.78±0.15 | -1.9±0.95 | -9.4±1.5 |
| **5** | 13±0.75 | -9.5±0.15 | -8.1±0.40 | -6.1±2.4 | -2.3±1.4 | -4.8±3.4 | -2.4±1.6 | -2.3±0.95 | -1.5±0.85 |
| **6** | 11±1.9 | -7.5±0.30 | 2.5±1.20 | -7.6±3.9 | -1.1±0.40 | -2.1±1.9 | -14±1.5 | -1.7±0.80 | -3.9±2.5 |
| **7** | 9.8±0.050 | -10±1.6 | -4.4±1.6 | -4.2±1.5 | -2.1±1.6 | -3.0±1.3 | -1.5±1.3 | -7.6±1.1 | -5.7±4.5 |
| **8** | 36±3.5 | -12±1.5 | 16±8.4 | -4.4±5.3 | -1.7±2.3 | -8.4±9.7 | -1.4±2.5 | -9.2±1.7 | -5.8±1.6 |
| **9** | 39±1.1 | -5.4±1.3 | 73±0.85 | 20.0±2.7 | -1.2±0.75 | -3.9±9.2 | -8.4±0.80 | -3.4±2.2 | -3.2±1.1 |
| **10** | 37±1.3 | 3.7±2.1 | 61±2.7 | -3.0±5.8 | -9.7±1.7 | -1.6±7.0 | -1.7±1.0 | 0.53±3.5 | -3.0±1.5 |
| **11** | -10±2.7 | 6.3±2.4 | -10±2.4 | 9.7±1.3 | 2.2±2.8 | -6.0±1.2 | -0.17±2.5 | 6.0±2.1 | 6.1±1.7 |
| **12** | -7.7±2.3 | 3.6±1.2 | -3.9±0.56 | 10±7.3 | 2.7±1.4 | -1.0±1.4 | 1.2±2.1 | 3.9±2.5 | 7.1±0.83 |
| **Ciprofloxacin** | 79.±0.3 | 75±2.8 | 83±0.40 | 76±0.50 | 79±0.35 | 77±0.65 | 87±0.15 | 71±0.050 | 80±0.95 |

**Table S3.** Inhibition rates of *A. salmonicida* of compounds **9** and **10**.

| Concentration (μM) | **9** | **10** | **Ciprofloxacin** |
|---|---|---|---|
| 100 | 67±1.5 | 71±0.47 | 81±1.7 |
| 50 | 75±0.93 | 79±0.47 | 84±1.8 |
| 25 | 68±9.31 | 23±1.7 | 83±2.4 |
| 12.5 | 30±2.8 | 21±4.1 | 84±1.9 |
| 6.25 | 21±0.0 | 11±1.3 | 37±2.8 |
